# Different genotypes of *Trypanosoma cruzi* produce distinctive placental environment genetic response in chronic experimental infection

**DOI:** 10.1371/journal.pntd.0005436

**Published:** 2017-03-08

**Authors:** Natalia Anahí Juiz, María Elisa Solana, Gonzalo Raúl Acevedo, Alejandro Francisco Benatar, Juan Carlos Ramirez, Priscilla Almeida da Costa, Andrea Mara Macedo, Silvia Andrea Longhi, Alejandro G. Schijman

**Affiliations:** 1 Grupo de Biología Molecular de la Enfermedad de Chagas (LaBMECh), Instituto de Investigaciones en Ingeniería Genética y Biología Molecular ‘‘Dr. Héctor N. Torres” (INGEBI-CONICET), Buenos Aires, Argentina; 2 Departamento de Microbiología, Parasitología e Inmunología, Instituto de Investigaciones en Microbiología y Parasitología Médica (IMPaM), Facultad de Medicina, Universidad de Buenos Aires (UBA), Buenos Aires, Argentina; 3 Departamento de Bioquímica e Inmunologia, Instituto de Ciências Biológicas, Universidade Federal de Minas Gerais, Belo Horizonte, Minas Gerais, Brazil; National Institute of Parasitic Diseases, CHINA

## Abstract

Congenital infection of *Trypanosoma cruzi* allows transmission of this parasite through generations. Despite the problematic that this entails, little is known about the placenta environment genetic response produced against infection. We performed functional genomics by microarray analysis in C57Bl/6J mice comparing placentas from uninfected animals and from animals infected with two different *T*. *cruzi* strains: K98, a clone of the non-lethal myotropic CA-I strain (TcI), and VD (TcVI), isolated from a human case of congenital infection. Analysis of networks by GeneMANIA of differentially expressed genes showed that “Secretory Granule” was a pathway down-regulated in both infected groups, whereas “Innate Immune Response” and “Response to Interferon-gamma” were pathways up-regulated in VD infection but not in K98. Applying another approach, the GSEA algorithm that detects small changes in predetermined gene sets, we found that metabolic processes, transcription and macromolecular transport were down-regulated in infected placentas environment and some pathways related to cascade signaling had opposite regulation: over-represented in VD and down-regulated in K98 group. We also have found a stronger tropism to the placental organ by VD strain, by detection of parasite DNA and RNA, suggesting living parasites. Our study is the first one to describe in a murine model the genetic response of placental environment to *T*. *cruzi* infection and suggests the development of a strong immune response, parasite genotype-dependent, to the detriment of cellular metabolism, which may contribute to control infection preventing the risk of congenital transmission.

## Author summary

Congenital transmission of *Trypanosoma cruzi*, the causative agent of Chagas disease, remains a problem of global public health impact in endemic areas where vectorial and transfusional transmission have been controlled and in non-endemic countries due to migration movements. Little is known about how the parasite´s presence and genetic variability affect placental capacity to protect the fetus. This study explores, for the first time, the effects of placental environment infection by analyzing parasite persistence and gene expression using functional genomics and biological network analyses in mice infected by two strains of *T*. *cruzi*, with differential capacity of congenital transmission. The infection with the strain with a stronger placental tropism was associated to a higher degree of up-regulation in genes related to innate immunity and response to interferon-gamma. Our findings suggest that the placental environment exerts a strong immune response in detriment of cellular metabolism modulated by the parasite strain. These findings constitute a significant contribution to better understand the mechanisms causing congenital infection of *T*. *cruzi*.

## Introduction

Maternal–fetal transmission of *Trypanosoma cruzi*, the etiological agent of Chagas disease, remains a public health problem that allows uncontrolled transmission of parasites from one generation to another, in endemic and non-endemic regions. Certainly, cases of congenital *T*. *cruzi* infection have been described in Japan, USA and Europe, especially in Spain [1, 2]. The risk of transmission is higher in the acute phase because of the great number of circulating parasites, but it may occur at any phase of the maternal disease [3]. Congenital infection has been associated with an increased risk of premature delivery, low birth weight, and more premature ruptures of the amniotic membranes, effects that may be related to placenta inflammation [4]. Studies in mice have reported also infertility and fetal growth retardation, associated or not with congenital infection, in both chronic and acute phase [5, 6, 7].

*T*. *cruzi* has been classified in six different discrete typing units (DTUs), named TcI to TcVI, according to biological, biochemical and genetic diversity [8]. Each DTU is formed by several parasite strains which are related to each other based on common molecular markers, but different to strains from other DTUs. These molecular markers currently used to define the *T*. *cruzi*-DTUs do not focus on the genes responsible for congenital transmission or pathogenicity of the parasite. In addition, except TcIV, the other five DTUs have been identified in human cases of congenital *T*. *cruzi* infection [3, 9]. Thus, the different genotypes of *T*. *cruzi* and their population characteristics, as parasite pathogenicity, virulence and tissue tropism may play an important role in congenital infection. However, until now it has not been found differences in the distributions of congenital cases and their respective parasite populations [3, 10–13]. Same genotypes are found in mothers and their infected newborns [11, 12, 14], although it was found a natural selection at clonal level in the parasite populations transmitted to the newborns [10, 12–15]. In murine models, previous studies comparing two different strains (K98 and RA, belonging to TcI and TcVI, respectively) showed differences in inflammatory compromise of the genital tract, the outcome of pregnancy and transmission of congenital infection [16]. Therefore, even though available data in humans suggested no association between particular *T*. *cruzi* genotypes with congenital infection, these findings cannot be omitted and deserve further study.

Because little is known about the genetic response to the infection by the most important barrier that *T*. *cruzi* faces to reach the fetus, the placenta, studies in infected mice might be a suitable model for understanding the potential role of *T*. *cruzi* genotypes on pregnancy and congenital infection.

Functional genomics is an important tool to study host-pathogen interactions, since it gives insight into the molecular mechanisms that control the onset of disease. The present study employed a transcriptomic approach combined with biological network analysis to highlight the differences between the responses of murine placenta environment to infection by two different *T*. *cruzi* strains, K98 a clone of the non-lethal myotropic CA-I strain, and VD (TcVI), a strain isolated from a human case of congenital infection.

## Methods

### Ethics statement

Animal care was in accordance with institutional guidelines of the “Asociación Argentina para la Ciencia y Tecnología de Animales de Laboratorio” (AACyTAL) and the project was approved by the “Comité Institucional para el Cuidado y Uso de Animales de Laboratorio” (CICUAL) of the School of Medicine, University of Buenos Aires, *Resol* 2426/2015.

### *T*. *cruzi* stocks

Two *T*. *cruzi* stocks were used: the myotropic clone K98 (TcI), a subpopulation derived from the CA-I strain, previously described by Mirkin [17] and, the monoclonal strain VD (TcVI) isolated by Risso from a case of congenital Chagas' disease [18]. These two strains have polar phenotypes: VD is more virulent and reaches its peak of parasitemia at 18–25 after infection whereas K98 displays a mild infection and its parasitemia peak is slightly delayed. Moreover, VD shows a slender shape in the bloodstream stage while K98 shows a stumpy shape [18]. DTU identification was done following the methodology reported by Burgos and coworkers [12] and monoclonality was verified by analysis of microsatellite loci as described Valadares and coworkers [19].

### Experimental approach and sample collection

C57Bl/6J mice were supplied by the animal facilities of Facultad de Ciencias Exactas y Naturales and were housed at the Department of Microbiology, School of Medicine, University of Buenos Aires, respectively. To obtain chronically infected mice, 4 inbred females between 6 and 8 weeks old were inoculated by the intradermoplantar (idp) route with 500 bloodstream trypomastigotes of K98 or with 50 bloodstream trypomastigotes of VD, because higher inocula of VD are lethal for this mouse strain by the intraperitoneal route [18]. Parasitemia was measured weekly by counting in a Neubauer chamber the number of parasites obtained from the tail vein blood diluted (1/10) in red blood cell lysis solution (Tris–NH4 Cl 0.83% pH 7.2). Chronic phase was considered when no parasites were detected in chamber (around 3 weeks post-infection). At day 30 post-infection two females were housed with 1 uninfected male per cage. To assess the day of fertilization, the vaginal plug was macroscopically searched [16], being the day of vaginal plug appearance considered as day 0.5 of pregnancy. A third group of 4 uninfected females of the same age and weight were used as control.

On day 18.5±1 of pregnancy, dams were euthanized and samples of maternal blood were taken by cardiac puncture and stored in EDTA solution until processed. The fetuses were withdrawn and samples from skeletal muscle were conserved at −80°C, for DNA extraction and qPCR analysis.

Finally, entire placentas from the naturally mated crosses were removed, thoroughly washed with sterile PBS and placed in RNAlater solution (Applied Biosystems, Foster City, CA) until used. As traces of maternal blood and other tissues such as decidua cannot be discarded as part of the samples, we have termed them as placental environment.

### Microarray

We analyzed total RNA from placental environments (the most proximal placentas from uterine horn of each dam were selected) for global gene expression via the Illumina array platform and using the mouse WG-6 v2.0 Expression BeadChip, service provided by Macrogen, Seoul, Korea.

### Microarray data analysis

Two approaches were performed, the Over-Representation Analysis and the Gene Set Enrichment Analysis [20]. Statistical significance of the expression data was determined using LPE test and fold change (FC) in which the null hypothesis was that no difference exists among groups. False discovery rate (FDR) was controlled by adjusting p value using Benjamini-Hochberg algorithm. Gene-Enrichment and Functional Annotation analysis for significant probe list was performed using DAVID (http://david.abcc.ncifcrf.gov/home.jsp). The free and open-source gene function prediction service, GeneMANIA (http://www.genemania.org), was used along with the widely used large-scale network visualization and integration tool, Cytoscape [21] to formulate and visualize the resultant integrated gene network. A gene set analysis using the GSEA package Version 2.0 [22, 23] from the Broad Institute (MIT, Cambridge, MA) was also used to analyze the pattern of differential gene expression between the two groups. Gene set permutations were performed 1000 times for each analysis. The normalized enrichment score (NES) was calculated for each gene set. GSEA results with a nominal p < 0.05 were considered significant. Biological process of GO was the pathway database used (http://geneontology.org/).

### Microarray validation

To validate the results of the microarray, two placentas from each dam (one already used in the microarray assay) were analyzed by means of RT-qPCR, as described below (total sample size = 8 placentas/ group)”. The number of genes analyzed and the selection criteria followed the recommendations previously described [24, 25] in microarray analyses in order to obtain a good correlation between microarray findings and RT- qPCR data: 14 genes exhibiting at least 1.4 fold change and a p-value ≤ 0.0001 (*Ccl3*, *Ccl4*, *Ccl7*, *Cd274*, *Cd3d*, *Cd8b1*, *Edn2*, *Gbp2*, *Gbp3*, *Gzmd*, *Igtp*, *Irgb10*, *Irgm1*, *H2-Aa*, *H2-Eb1*). *Cxcl1* and *S100a9* were chosen because these genes were among the few ones up-regulated in K98 group and *Gbp6* because it is involved in the secretory granule pathway. The expression levels were normalized to *Gapdh*.Primer sequences are listed in S1 Table. Moreover, in order to test expression at protein level, Western Blot was performed for CXCL1 and CD274 proteins. Briefly, placentas were homogenized in buffer 50 mM Tris-HCl, 150 mM NaCl, pH 7.4 (TBS) in the presence of 0.1% SDS and a protease inhibitor kit (Complete Mini EDTA-free, Roche, Germany). The homogenates were centrifuged at 17,000×g for 10 minutes at 4°C to remove insoluble material. Protein concentration of supernatants was determined by Bradford reagent (Sigma-Aldrich, St. Louis, MO, USA) and equal amount of protein (100 μg per lane) was resolved on a 10% SDS-PAGE. After blotting and blocking of nitrocellulose membranes with TBS, 0.1% Tween 20 (TBS-T) 3% BSA, they were incubated overnight at 4°C with rabbit anti-CXCL1 polyclonal Ab 1:2000 (PAI-2920, Thermo Fischer, Rockford. IL, USA) or rat anti-CD274 monoclonal Ab 1:1000 (10F.9G2, Bio X Cell, West Lebanon, NH, USA) in TBS-T 1% BSA. Blots were then incubated with horseradish peroxidase-conjugated anti-rabbit IgG 1:3000 (Vector Labs, UK) or horseradish peroxidase-conjugated anti-rat IgG 1:3000 (Sigma-Aldrich, St Louis, MO, USA), respectively. Immunoreactive proteins were revealed by enhanced chemiluminescence (Pierce ECL-Plus, Thermo Scientific, Rockford, IL, USA) according to manufacturer’s instructions. The bands were scanned and quantified using ImageJ software (version 1.410).

To reprobe same membranes with rabbit anti-GAPDH monoclonal Ab 1:5000 (14C10, Cell Signalling, Boston, MA, USA) as a loading control, the membranes first were incubated in stripping buffer (200 mM Glycine-HCl pH 2.2, 0.1% SDS, 1% Tween 20) twice for 10 minutes at room temperature while shaking, washed with TBS-T, and then blocked and immunodetected as describe above.

### Determination of *T*. *cruzi* loads and characterization

DNA from maternal blood, placental and fetal tissues was extracted using the High Pure PCR Template Preparation kit (Roche Diagnostics Corp., Indiana, USA) following manufacture instructions.

Total RNA was extracted from placentas using the TRIzol reagent (Invitrogen, Carlsbad, CA) according to manufacturer's instructions, treated with RQ1 RNase-Free DNase (Promega, Madison, USA) and stored at −80°C until used. RNA purity and integrity were evaluated by ND-1000 Spectrophotometer (NanoDrop, Wilmington, USA), Agilent 2100 Bioanalyzer (Agilent Technologies, Palo Alto, USA).

Determination of Satellite *T*. *cruzi* DNA loads in maternal, placental and fetal samples was performed by means of quantitative Real Time PCR, as previously reported [26] and normalized with murine *β-Actin* DNA (housekeeping gene) in the same sample (β-ACT Fw: 5´ CGGAACCGCTCATTGCC 3´ and β-ACT Rv: 5´ ACCCACACTGRGCCCATCTA 3´). Results were expressed as relative amplification respect to that of *β-Actin* DNA fragment.

Detection of Satellite *T*. *cruzi* DNA in fetal samples was performed by qualitative Real Time PCR [26] without using a standard curve for quantification.

The presence of viable parasites in placental tissue was evaluated by amplification of 18S *T*. *cruzi* RNA. For this purpose, 1 μg of RNA per sample was reversed transcribed using SuperScript II Reverse Transcriptase kit and random primer (Life technologies, Ontario, Canada). Reactions for quantitative reverse transcription PCR (RT-qPCR) were prepared with 0.15 μM forward and reverse primers (18S Fw: 5´ TGGAGATTATGGGGCAGT 3´ and 18S Rv: 5´ GTTCGTCTTGGTGCGGTCTA 3´), 1X FastStart Universal SYBR Green Master (Roche Diagnostics Corp., Indiana, USA) and each of the diluted template cDNAs (1:100 in DNAse free water). RT-qPCR was analyzed on the Applied Biosystems 7500 Real-Time PCR System using following cycling conditions, as recommended by the manufacturer (Applied Biosystem, California, USA): 95°C 10 min; 5 cycles of 95°C for 15 sec and 64°C 1 min, 35 cycles of 95°C for 15 sec and 63°C 1 min; melt from 60 to 95°C rising at 0.2°C per second. *Gapdh* (glyceraldehyde-3-phosphate dehydrogenase) was used as internal control to normalize mRNA levels using the following primers: GAPDH Fw: 5´ ACTCCCACTCTTCCA 3´ and GAPDH Rv: 5´ TCCACCACCCTGTTG 3´ and the standard curve method was applied.

### Analysis of minicircle signatures and microsatellite loci polymorphism

Restriction fragment length polymorphism (RFLP)-PCR profiling was performed as described by Burgos [12] with 1 μg of purified minicircle amplicons that were digested with 1 U of MspI + RsaI + HinfI restriction enzymes for 4 h at 37°C. The digestion products were visualized after 10% PAGE and SYBR Gold Nucleic Acid Gel Stained (Invitrogen, Eugene, Oregon). Markers of 10 and 25 bp, DNA Step Ladder (Promega, Madison, USA), were included in runs.

Full nested-PCR targeted to sequences flanking microsatellite repeats for the loci TcTAC15, TcATT14, TcGAG10, TcCAA10 and TcTAT20, were carried out as previously described [19]. Each PCR was performed in a total volume of 15 μL using 1 U of Taq Platinum DNA Polymerase (Invitrogen, Carlsbad, CA). The first PCR rounds were carried out using 2 μL of DNA obtained from blood and placental tissue samples, whereas for the second PCR rounds 1 μL of the amplified products obtained in the first PCR round were used as DNA template. Amplifications were performed in a Veriti Thermal Cycler (Applied Biosystems, Foster City, CA). To determinate the allele sizes, 0.5 μL of PCR fluorescent products were analyzed in 6% denaturing polyacrylamide gels of an ALF DNA sequencer (GE Healthcare, Milwaukee, WI) and compared with fluorescent DNA fragments of 75–320 bp using Allelelocator software (GE Healthcare).

### Statistics

Statistical analyzes were performed using GraphPad Prism 6.01 and InfoStat version 2015. (http://www.infostat.com.ar). Mann-Whitney test and Kruskal-Wallis with multiple comparisons were employed for comparison between two groups and among three groups, respectively.

### Accession numbers

The data discussed in this publication have been deposited in the National Center for BiotechnologyInformation (NCBI) Gene Expression Omnibus (GEO) and are accessible through GEO Series accession number GSE85996 (http://www.ncbi.nlm.nih.gov/geo/query/acc.cgi?acc=GSE85996).

## Results

### Analysis of differential placental gene response to *T*. *cruzi*

We performed a microarray analysis to screen for genes whose expressions were altered in the placental environment upon infection with K98 or VD strains. Two different approaches were faced in order to determine the pathways involved. The first approach was “Over-Representation Analysis”, intended to detect protein and gene interactions. This analysis statistically evaluates the fraction of genes of a particular pathway among the complete set of genes that show changes in expression. The second approach used was “Gene Set Enrichment Analysis” (GSEA), which detects small changes in predetermined gene sets, in order to identify significant enrichment pathway-level effects. It was used to perform Gene Ontology (GO) analysis with those gene sets derived from the “Biological Process Ontology”.

#### Over-representation analysis

**Fig 1:**
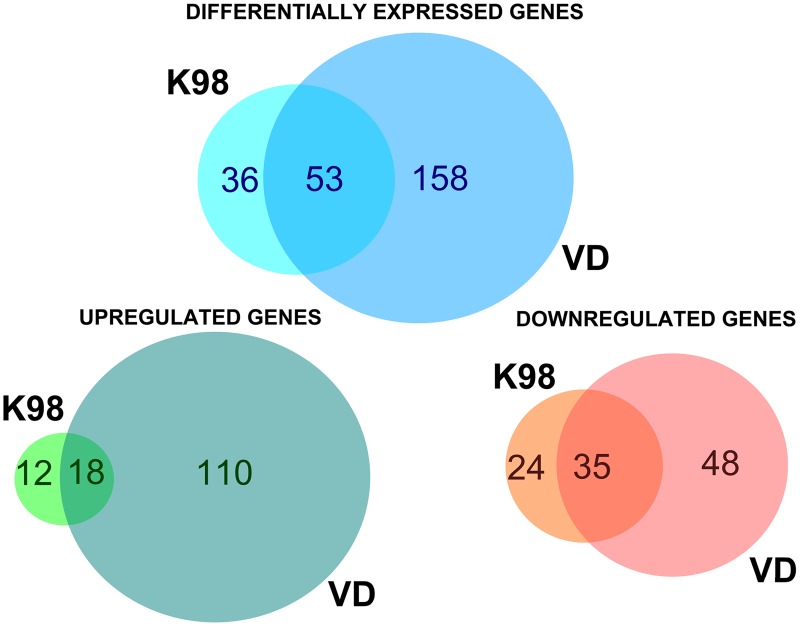
Venn diagrams analysis. Numbers embedded in each complete circle denote the total numbers of DEGs of the indicated infected groups in comparison to the uninfected control group; those in the overlapping region depict the number of shared genes between the two conditions.

The microarray analysis from C57Bl/6J mice between infected groups and the uninfected control group rendered a total of 247 differentially expressed genes (DEGs), with a fold change |FC| ≥ 1.5 and a significance level ≤ 0.05 (S2 Table). Out of them, 140 genes were up-regulated and 107 were down-regulated (Fig 1, S3 and S4 Tables). The greatest fold differential expressions encountered were a 17-fold up-regulated *Cxcl9*, a T-cell chemoattractant which is induced by IFN-γ, inVD and a 4-fold down-regulated *Tph1* gene, which codifies for an isoenzyme of tryptophan hydroxylase in K98.

A stronger response to infection was observed in those placental environments from animals infected with VD strain, with 211 DEGs, than in those infected with K98, with only 89 DEGs (Fig 1). Fifty three DEGs compared with the control group were shared between both infected groups (Fig 1). However, there was an opposite effect in animals infected by both tested strains: while 69% (59/89) of genes were down-regulated by K98 infection, VD was able to produce this effect on 39% (83/211) of them compared with uninfected animals.

**Fig 2:**
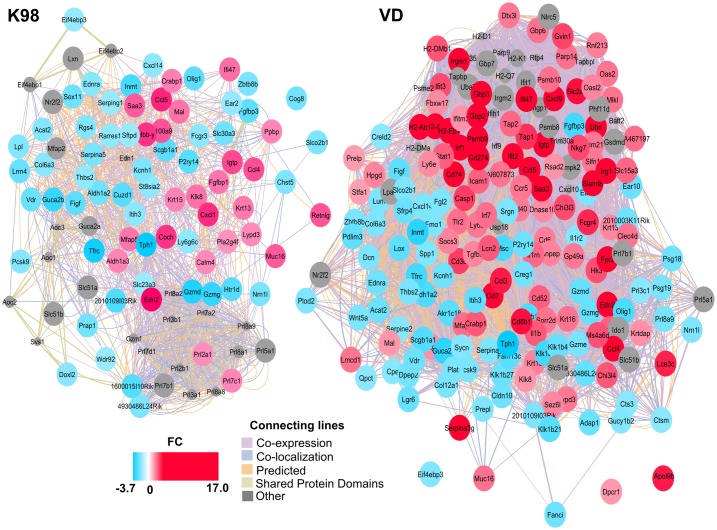
The interaction networks for DEGs between K98 and VD infected groups compared with the control groups predicted by GeneMania and visualized in Cytoscape. Genes/proteins are depicted as colored circles and indicated by the color scheme, where shades in red correspond to FC > 1.5, shades in light blue correspond to FC < -1.5 and gray circles are other genes/proteins related to these DEGs, but not found in the microarray. Experimentally detected associations between genes/proteins are shown as connecting lines.

Analysis using GeneMANIA function prediction service was performed along with the widely used large-scale network visualization and integration tool, Cytoscape, in order to formulate and visualize the resultant integrated gene network with genes with |FC| ≥ 1.5. The co-localization, co-expression, shared pathways, protein domains and genetic, physical and predictive interactions of DEGs in K98 and VD infected groups compared with the control group are shown in Fig 2.

**Fig 3:**
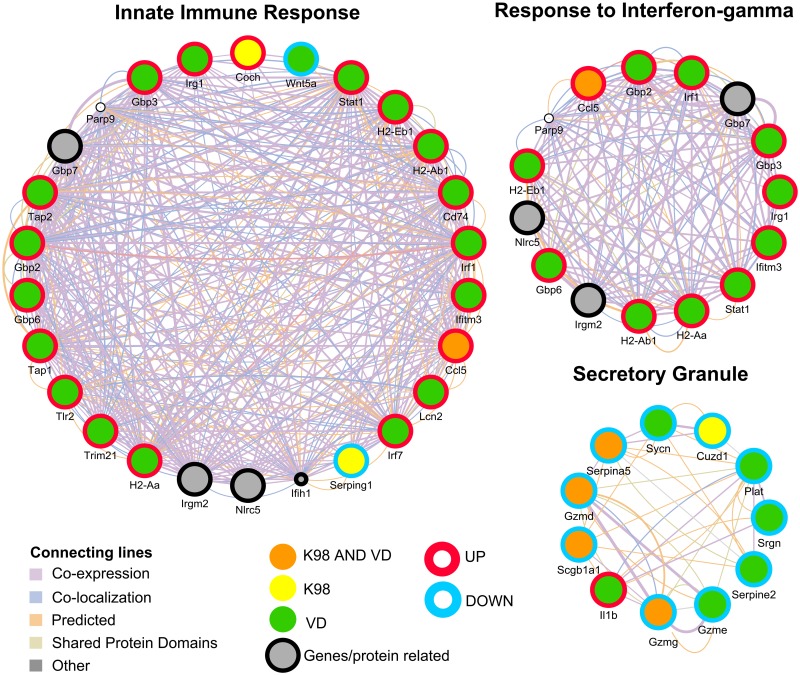
Visualization of three networks for DEGs between K98 and VD infected groups compared with the control group as predicted by GeneMania: “Innate Immune Response”, “Response to Interferon-gamma” and “Secretory Granule”. Genes/proteins are depicted as colored circles, where yellow indicates a DEG in K98 group, green in VD group and orange in both K98 and VD groups. The color of border lines indicates up-regulation (red) or down-regulation (light blue) and gray circles are other genes/protein related but not found in the microarray. Experimentally detected relationships between genes/proteins are shown as connecting lines.

Among the networks predicted by GeneMania, Fig 3 illustrates the “Secretory granule”, “Innate Immune Response” and “Response to Interferon-gamma”. The “Secretory Granule” was a down-regulated pathway respect to control in both VD and K98 groups (q-value = 0.02). *Serpina5*, *Gzmd*, *Gzmg* and *Scgb1a1* are genes which transcription was negatively affected by the infection by both strains. Only a single gene related to this network encoding *Il-1β* was up-regulated in VD group related to the uninfected animals. IL-1β is produced in activated macrophages and is an important mediator of the inflammatory response involved in a variety of cellular activities, including cell proliferation, differentiation, and apoptosis. The gene *Cuzd1*, which has been found to play a role in the uterus during late pregnancy, was the only gene down-regulated in K98 but not in VD when compared to controls (Fig 3).

“Response to Interferon-Gamma” (q-value = 2.40 x 10^−2^) and “Innate Immune Response” (q-value = 4.90 x 10^2^) were detected after analysis of DEGs in VD but not in K98 group (Fig 3).

Moreover, “Response to Interferon-Beta” (q-value = 3.17 x 10^−3^) and “Antigen Processing and Presentation” (q-value = 1.85 x 10^−4^) among others were also detected in VD but not in K98 group in the comparison against the control group. IFN-γ and IFN-β and Innate Immune Response pathways shared *H2-Aa*, *H2-Ab1*, *H2-Eb1* genes, while networks of response to IFN-γ and IFN-β shared additional genes: *Gbp2*, *Gbp3*, *Gbp6*, *Irf1*, *Irg1*, *Ifitm3* and *Stat1*. Considering the network of response of IFN-γ, all eleven genes up-regulated in VD group displayed a higher transcription FC compared with the uninfected mice. The only gene with positive FC related to IFN-γ response in K98 group was *Ccl5* (FC = 3.11, p < 0.0001); however this FC increased to 8.57 (p < 0.0001) in VD, showing a clear stronger effect of this strain in the latter.

Furthermore, the “Innate Immune Response” network showed an up-regulation of most genes in VD group (Fig 3). Interestingly, two genes had a negative rate of transcription respect to control: the gene *Wnt5a* in VD group (FC = -1.80, p = 0.010), which is implicated in several developmental processes, including regulation of cell fate and patterning during embryogenesis and the gene *Serping1* in K98 group (FC = -1.63, p = 0.040). This gene encodes a highly glycosylated plasma protein involved in the regulation of the complement cascade.

#### Gene Set Enrichment Analysis (GSEA)

For this approach two parameters were taken into account, 1) The Normalized Enrichment Score (NES): the degree to which each gene set is over-represented, comparing each infected group (K98 and VD) with the uninfected control group at the top or bottom of the ranked list of genes in the expression dataset, after being normalized across analyzed gene sets; and 2) False Discovery Rate (FDR) q-value: the estimated probability that the NES may represent a false positive finding.

**Fig 4:**
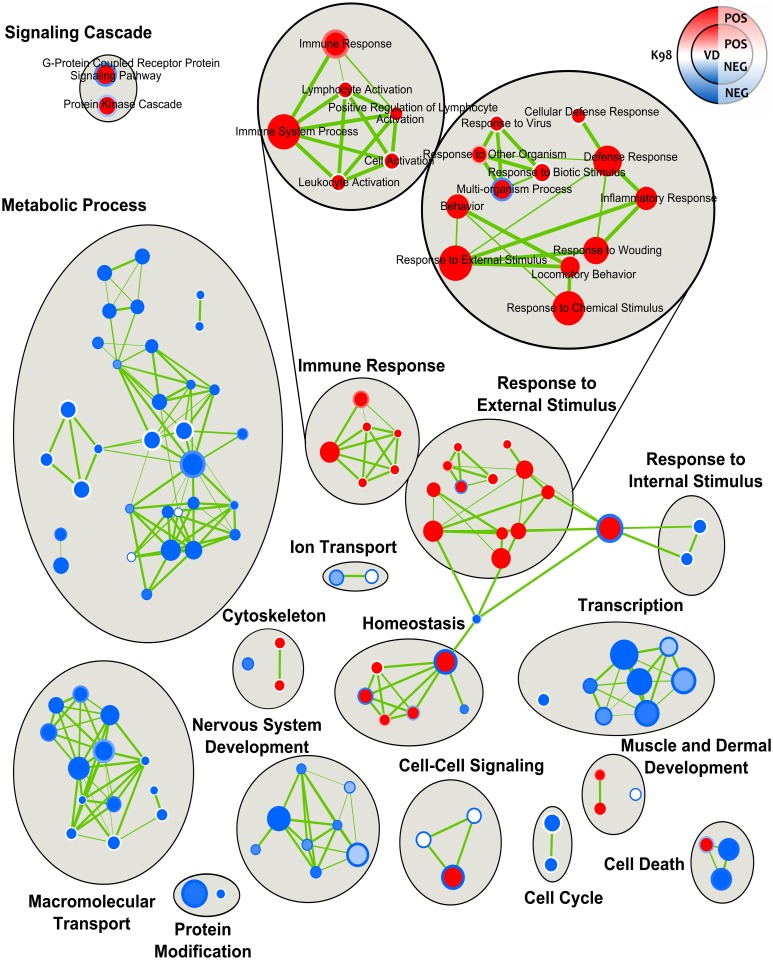
Enrichment map of the placental environment response to infection by *T*. *cruzi*. The Enrichment Map arranges enriched gene-sets as a similarity network, where nodes correspond to gene-sets and links correspond to overlap of member genes. The network was manually rearranged to improve layout, and major clusters were manually labeled. Predicted pathways are depicted as circles, where shades in red correspond to up-regulated gene-sets and shades in light blue correspond to down-regulated gene-sets. The filling color corresponds to VD group and the border line color to K98 group.

Several pathways involved in metabolic processes and macromolecular transport were found to be down-regulated in both infected groups (Fig 4, S5 and S6 Tables). Among metabolic processes, the three following ones were down-regulated in both K98 and VD infected groups: “Lipid Metabolic Process” (NES = -3.79 and -3.42, respectively; FDR.q.val <0.0001), “Carbohydrate Metabolic Process” (NES = -3.55 and -3.46, respectively; FDR.q.val <0.0001) and “Proteolysis” (NES = -2.96 and -2.86, respectively; FDR.q.val <0.0001). With respect to macromolecular transport, “Intracellular Transport” was one of the down-regulated processes in both infected groups (K98: NES = -1.99 and FDR.q.val = 0.003; VD: NES = -2.8 and FDR.q.val = 0.0001). Transcription was also affected negatively by *T*. *cruzi* infection, being some of the most representative pathways “Regulation of Transcription DNA-Dependent” (K98: NES = -2.67 and FDR.q.val = 0.001; VD: NES = -2.01 and FDR.q.val = 0.022) and “Regulation of RNA Metabolic Process” (K98: NES = -2.67 and FDR.q.val = 0.001; VD: NES = -2.13 and FDR.q.val = 0.013).

Moreover, a negative regulation of cell death processes was observed in both infected groups with two pathways down-regulated: “Regulation of Programmed Cell Death” (K98: NES = -1.80 and FDR.q.val = 0.057; VD: NES = -2.18 and FDR.q.val = 0.011) and “Regulation of Apoptosis” (K98: NES = -1.80 and FDR.q.val = 0.058; VD: NES = -2.20 and FDR.q.val = 0.010).

A markedly positive regulation of “Immune System Process” was associated with infection by both strains (K98: NES = 2.05 and FDR.q.val = 0.049; VD: NES = 4.15 and FDR.q.val = 0.0001). However, the immune response was stronger in VD group showing up-regulation of the pathways “Leukocyte Activation” (NES = 2.48 and FDR.q.val = 0.006), “Cell Activation” (NES = 2.43 and FDR.q.val = 0.008) and “Lymphocyte Activation” (NES = 2.38 and FDR.q.val = 0.009). For “External Stimulus”, an opposite result was observed in “Multi-organism Process”, up-regulated in VD (NES = 2.70 and FDR.q.val = 0.002) and down-regulated in K98 (NES = -1.77 and FDR.q.val = 0.065). “Cellular Defense Response” was also a pathway overrepresented in VD (NES = 3.29 and FDR.q.val <0.0001) but not in K98.

Certain pathways related to homeostasis were differentially represented between infected groups. For example, NES for “Homeostasis Process” was negative in K98 (-1.85 and FDR.q.val = 0.05), whereas it was positive in VD (2.41 and FDR.q.val = 0.0085). Other gene sets with differential representation between infected groups were those related to signaling pathways: “G-Protein Coupled Receptor Protein Signaling Pathway” (K98: NES = -2.02 and FDR.q.val = 0.027; VD: NES = 2.683 and FDR.q.val = 0.003) and “Protein Kinase Cascade” (K98: NES = -1.55 and FDR.q.val = 0.146; VD: NES = 2.338 and FDR.q.val = 0.010). Finally, the biological process “Cell-Cell Signaling” (K98: NES = -2.42 and FDR.q.val = 0.004; VD: NES = 2.46 and FDR.q.val = 0.007), as well as “Negative Regulation of Developmental Process” (K98: NES = -1.62 and FDR.q.val = 0.114; VD: NES = 2.22 and FDR.q.val = 0.016) were down-regulated in K98 but up-regulated in VD.

#### Microarray validation

To confirm the findings from the microarray assay, a subset of 18 genes were selected for validation at the mRNA and/or protein levels. RT-qPCR analysis (S1 Fig) demonstrated that infection with both strains of *T*. *cruzi* resulted in increased expression of *Ccl4* and *Edn2* genes compared with control (p = 0.0014 and p = 0.0111, respectively), whereas *Gzmd* was down-regulated in both infected groups (p = 0.0047). *Cxcl1* and *S100a9* were up-regulated in K98 but not in VD infected samples when normalized to control group (p = 0.0413 and p = 0.0074, respectively). Consistently with the microarray data, the following genes were up-regulated in VD but not in K98 groups, when the expression levels were normalized to control group: *Cd3d* (p≤ 0.0001), *Cd8b1* (p = 0.0005), *Gbp2* (p = 0.0009), *Gbp3* (p = 0.0249), *Gbp6* (p = 0.0052), *H2-Aa* (p = 0.0019), *H2-Eb1* (p = 0.0039), *Igtp* (p = 0.0002), *Irgb10* (p = 0.0023), *Irgm1* (p = 0.0005) genes. Although no differential expressions of *Ccl7* and *Cd274* was found using Mann-Whitney test (p = 0.1877 and p = 0.0648, respectively), Kruskal-Wallis analysis comparing the three groups showed a greater level of expression in VD group (p = 0.0070 and p = 0.0015, respectively) compared with the control.

Protein expression levels were also examined by Western blot for CXCL1 and CD274 proteins (S2 Fig). According to microarray and RT-qPCR experiments, a major expression of CXCL1 was observed in K98 group compared with VD (p< 0.01) and control groups (p< 0.05). Moreover, the expression level of this protein in VD infected mice was lower than in the control group, but this difference was not significant (S2 Fig).

In the other hand, the microarray assay could detect differences in VD but not in K98 in the expression of *Cd274* gene, however by Western blot and RT-qPCR, samples from both infected mice expressed comparable protein levels of CD274 and higher than control mice (p<0.01) (S2 Fig). These data confirmed that mRNA expression changes observed upon *T*. *cruzi* infection were associated with parallel changes at the protein level.

### Characterization of parasitic burden in placental environment vs maternal blood stream

**Fig 5:**
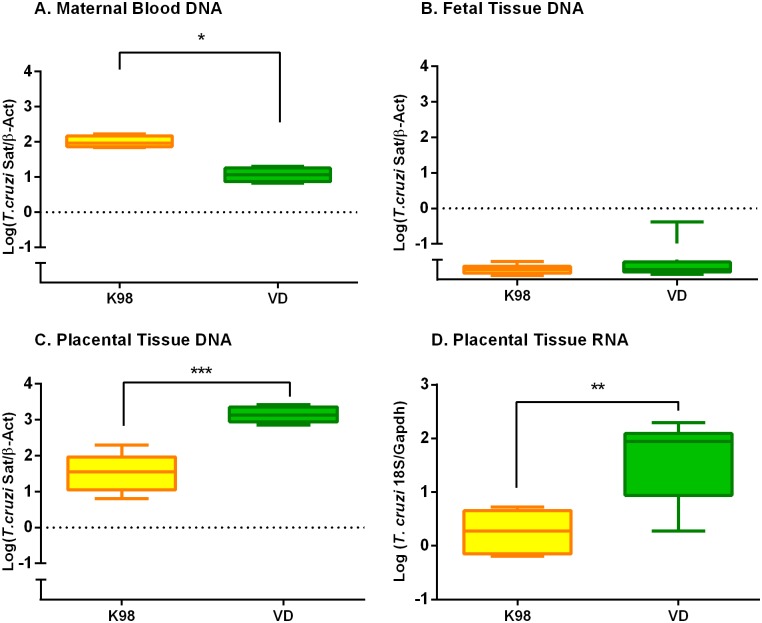
*T*. *cruzi* VD strain displayed a higher placental tropism than K98. The parasite load was quantified by Real Time amplification of Satellite DNA in: (A) maternal blood; (B) fetal tissues and (C) placental environment, normalized with mouse *β-Actin* DNA. (D) The detection in placental environment of *T*. *cruzi* gene expression was analyzed by RT-qPCR targeted to the parasite 18S RNA and normalized with mouse *Gapdh* RNA in K98 and VD groups. *** p < 0.0001; ** p < 0.01; *p < 0.05.

We aimed to find out whether the differential gene expression detected in the placental environment of infected animals with VD and K98 strains was associated with organ parasite persistence. Indeed, parasite burden in VD infected animals was two times higher (Log (median) = 3.134; Q_1_ = 3.04 and Q_3_ = 3.37) than those from K98 infected mice (Log (Median) = 1.554; Q_1_ = 0.94 and Q_3_ = 1.93) (p = 0.0002) (Fig 5c). The median logarithmic parasitic loads observed in maternal blood where 1.965 (Q_1_ = 1.84 and Q_3_ = 2.00) for K98 and 1.058 (Q_1_ = 0.82 and Q_3_ = 1.10) for VD (p = 0.029) (Fig 5a). In K98 group, the parasite load in dams´ blood compared with that detected in placental environment was approximately three times higher, whereas in VD group the increment was 120 times higher than in bloodstream, suggesting placental tropism of VD (p < 0.0001).

In order to elucidate if *T*. *cruzi* DNA detected in the placental environment corresponded to living parasites and not to mere DNA, RT-qPCR of *T*. *cruzi* 18S RNA gene was designed and performed in these samples (Fig 5d). RT-qPCR results were in agreement with those observed in DNA samples from infected mice; in fact the expression level of *T*.*cruzi* 18S RNA in these samples infected with VD were significantly higher (Log (Median) = 1.941, Q_1_ = 0.78 and Q_3_ = 2.06) than in K98 group (Log (Median) = 0.275, Q_1_ = -0.18 and Q_3_ = 0.49) (p = 0.002), confirming placental tissue preferential site for VD spreading.

**Fig 6:**
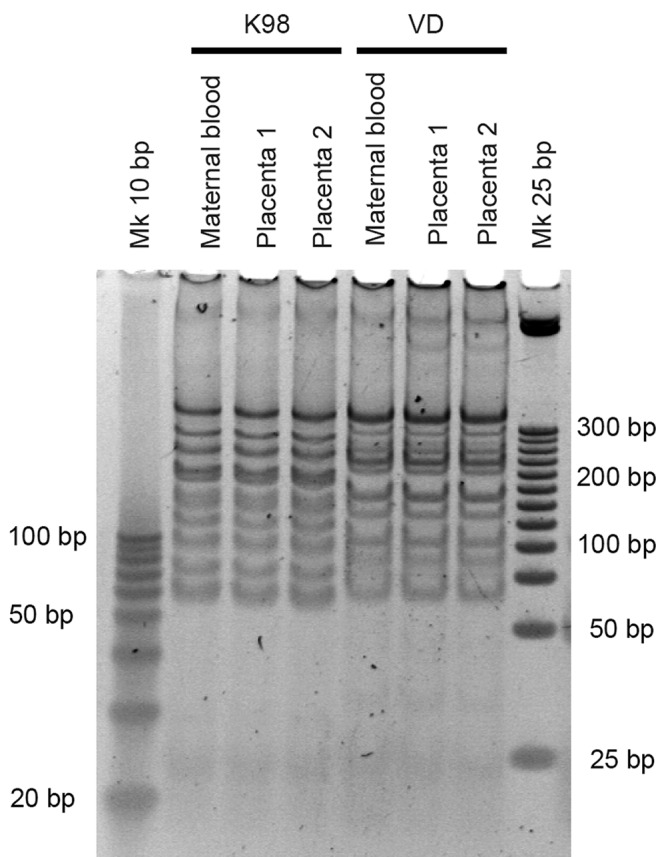
Examples of minicircle signatures by restriction fragment length polymorphism of maternal and placental parasite populations for K98 and VD groups. Minicircle signatures were visualized after 10% PAGE and SYBR Gold Nucleic Acid Gel Stained. Molecular weight markers of 10 and 25 bp were included.

Minicircle signatures were characterized in bloodstream and placental environment (Fig 6). They showed identical intra-group profiles with Jaccard’s coefficients (JD) of 0 for K98 and VD in all tested paired samples (n = 8 for K98 and VD), and clearly distinctive for each strain group (inter-group JD = 0.875). Microsatellite loci polymorphism analysis confirmed the monoclonality of the parasite strains and showed identical profiles between paired bloodstream-placental samples (S7 Table).

### PCR-based analysis of skeletal tissues from fetuses of infected dams

We analyzed by qualitative Real Time-PCR skeletal tissue samples from fetuses withdrawn from dams infected by both parasite stocks (N = 27 and N = 24 from K98 and VD groups, respectively), obtaining detectable amplification in all samples, though with Ct values between 35 and 40 in all samples, indicative of very low parasitic burden. Furthermore, the fetuses belonging to the eight placentas used in the microarray validation experiments were tested by qPCR (Fig 5b). These samples presented median logarithmic values of parasitic load significantly lower than those obtained in placental tissues and maternal blood of both infection groups (p< 0.001 for both groups and tissue types; Fig 5). These findings suggest contamination with DNA traces from maternal tissues rather than congenital infection.

## Discussion

Chagas disease is a problem of global public health impact and congenital (mother to child) infection is a remaining problem in endemic countries and the most common form of transmission in non-endemic regions. Host response against *T*. *cruzi* infection is still being investigated and little is known about how the presence of this parasite affects the placenta that usually represents a barrier for *T*. *cruzi* to reach the fetus during pregnancy. Several difficulties arise when studying the association between *T*. *cruzi* genotypes and risk of congenital infection and the placental genetic response: small sample volume of neonatal blood, small sample sizes (as a result of reduced transmission rate), low sensitivity of conventional diagnostic and genotyping methods, and deficient follow-up [27]. In the present study, we explored the effects of the infection in the placental environment with two different genotypes of *T*. *cruzi*, by analyzing gene expression and parasite persistence in a murine model using microarray analysis. Several genes were affected in their expression levels, up and down, in association to infection and more remarkably in VD group, perhaps as a result of the higher placental tropism displayed by this strain. The present study also combined this transcriptomic approach with biological network analyses to highlight the differences between the responses of murine placental environment to the infection with different *T*. *cruzi* genotypes.

Our first approach of Over-Representation Analysis evaluated the fraction of genes in a particular pathway found among DEGs with FC ≥ |1.5|. One of the down-regulated networks shared by both infected groups was the “Secretory Granule”, in which granzymes play a fundamental role. A down-regulation of these proteins has been observed in fetal NK cells of umbilical cords from newborns congenitally infected with *T*. *cruzi* as a mechanism of modulating the immune response [28]. These might be one of the multiple strategies to escape or modulate antigen presentation and T-cell-mediated anti-*T*. *cruzi* response of the host leading to intracellular parasite persistence during chronic infection. It has been described that the maternal CD8 T-cell response to placental antigens and to pathogen antigens are independent pathways [29], so the up-regulation of several genes that participate in events of antigenic processing and presentation in the placental environment during VD infection, might indicate that it displays all its weapons to defend the fetus from infection. The IFN-γ response, an essential mechanism to control survival and proliferation of intracellular pathogens, was another up-regulated network observed in VD group. Among the up-regulated genes involved in that network, three members of the guanylate binding protein family (GBPs), showed FCs higher than 2. Interestingly, although it is known that *T*. *cruzi* and *Toxoplasma gondii*, both intracellular protozoans and producers of congenital infections, have different mechanisms of cellular invasion, it has been reported that *T*. *gondii* produces a recruitment of GBPs in vacuoles and, each GBP has a specific role in resistance, also depending on the virulence of the strains involved [30, 31].

We have performed another approach with a hypothesis of functional class scoring, since great changes in individual genes may have significant effects on pathways but also weaker and coordinated changes in sets of functionally related genes (i.e., pathways) can have significant impact. Applying GSEA and using the dataset of Biological Process of GO, we found that metabolic processes as well as transcription and macromolecular transport were in general down-regulated in the placental environment of infected animals and these data are consistent with fetal growth retardation observed in mice from dams infected by *T*. *cruzi* [5, 32].

Mammalian cell invasion by *T*. *cruzi* requires cell signaling and the pathways induced may be different depending on the parasite strain [33]. It has been reported that inhibition of protein kinases significantly inhibits the infection of macrophages by *T*. *cruzi* [34] and also the pro-kineticin receptors in mammalian cells, which are G protein-coupled receptors [35]. Moreover, it has been described an activation of host protein kinase C by RA strain (TcVI) that favors infection [36]. In this study, we found that *T*. *cruzi* infection by different genotypes had a distinct effect on protein kinase cascade as well as on the signaling pathway of G-protein coupled receptor protein, up-regulated by VD and down-regulated by K98 infected samples. So, the negative regulation of these pathways could explain the lower levels of placental infection observed in K98 group. Likewise, positive regulation by VD could explain the found high parasite loads.

Another interesting finding of our analysis was the down-regulation of apoptosis observed in the placental environment of animals infected by both strains. This prevention of apoptosis might be a mechanism used by the parasite to persist all along infection, reducing potential damage, as observed in cardiomyocytes [37].

Different placental tropism of *T*. *cruzi* strains has been described [38]. Our results show that VD displays stronger placental tropism than K98 strain, revealed as an increased in the amount of parasitic burden and 18S RNA expression there compared with dams´ blood parasitic loads. It is worth mentioning that VD strain was isolated from a human case of congenital infection [18]. In dams infected with K98, higher levels of bloodstream parasites were observed at around 48 days post-infection compared with mice infected with VD. This is in agreement with findings in C3H/HeN female mice infected with K98 (44 days post-inoculation) that showed higher parasitemias than mice infected with RA [16]. No significant differences in parasite burden were observed for K98 parasites in dams´ blood and the placenta, suggesting poor tropism, in contrast to VD, demonstrated by both qPCR and RT-qPCR, although a higher duplication rate in VD might not be discard. Both K98 and VD stocks were monoclonal, so no differences in genetic constitution between parasite populations were detected in bloodstream and placental environment by means of minicircle signatures and microsatellite *loci* polymorphism analyses (Fig 6 and S7 Table).

In order to detect congenital infection, we analyzed by qPCR fetuses from dams infected by both parasite strains, obtaining very low parasitic burden in all tested samples from both infection groups, below maternal bloodstream and placental loads (Fig 5a and 5b). This was indicative of DNA contamination from maternal tissues rather than of true congenital infection. In fact, K98 has been demonstrated not to cause congenital transmission in mice [16]. In agreement with our findings, it has been reported that PCR positivity in pups close to delivery may not be reliable [7]. Besides, we have tested by qPCR one month-old pups born to dams infected with the VD stock, with negative results, demonstrating no congenital infection (unpublished results). Altogether, these observations are in agreement with previous studies showing that congenital transmission in mice is a very rare event; changes in placental gene expression driven by *T*.*cruzi* infection appear efficient to control the parasite, precluding congenital transmission.

The placenta is a complex environment where mother and fetus coordinately interact and, during an infection, the presence of the pathogen adds complexity by altering the immunoregulatory circuits. To our knowledge, the present study is the first one to describe the genetic response of this environment to *T*. *cruzi* infection and reveals host pathways leading to generate a strong immune response, in order to limit the risk of congenital infection, to the detriment of cellular metabolism. Moreover, we have found that this effect may be modulated by the parasite genotype. New studies to deepen insight in this host response, at both the genetic and protein level, are necessary to better understand the mechanisms causing congenital infection of *T*. *cruzi*.

## Supporting information

S1 Table Primers used in RT-qPCR for validation of microarray results. (PDF)

S2 Table *Fold change* and adjusted p-values from microarray analysis. (PDF)

S3 Table Genemania results for K98 group. (PDF)

S4 Table Genemania results for VD group. (PDF)

S5 Table Go-biological process with FDR.q.val < 0.05 found by GSEA analysis in K98 group. (PDF)

S6 Table Go-biological process with FDR.q.val < 0.05 found by GSEA analysis in VD group. (PDF)

S7 Table Examples of microsatellite identification of *T*. *Cruzi* isolates from maternal and placental populations in mice infected with K98 and VD genotypes. The table shows only the polymorphic loci.
(PDF)

S1 Fig Validation of microarray by RT-PCR. The expression levels of the selected genes were expressed as logarithms of fold change in arbitrary units (Log (FC)) normalized to *Gapdh*. Red symbols represent placenta samples used in microarray assay and black symbols represent independent samples. The line for each of the scatters represents the median value and significance obtained by Mann-Withney test was indicated as follows: **** p < 0.00001; *** p < 0.0001; ** p < 0.01; *p < 0.05.
(TIF)

S2 Fig Validation of microarray by western blot. Inmunoblot analysis of CXCL1 and CD274 in placentas samples from K98, VD and control groups. Inmunoreactive protein bands were quantified by densitometry. Results are expressed as arbitrary units (AU), related to GAPDH. Statistical analysis was performed by Kruskal-Wallis with multiple comparisons. ** p < 0.01; *p < 0.05.
(TIF)
