# Supplementary material for: Different genotypes of Trypanosoma cruzi produce distinctive placental environment genetic response in chronic experimental infection
Source: PLoS Negl Trop Dis. 2017 Mar 8;11(3):e0005436. doi: 10.1371/journal.pntd.0005436 (PMC5358786; doi:10.1371/journal.pntd.0005436)
Supplement: S1 Table — (PDF) [file pntd.0005436.s001.pdf]

S1 Table. Primers used in RT-qPCR for validation of Microarray results.

Gene Symbol	Refseq NM	Forward Primer Sequence (5'->3')	Reverse Primer Sequence (5'->3')
Ccl3	NM 011337.2	CCATATGGAGCTGACACCCC	TCAGGAAAATGACACCTGGCT
Ccl4	NM 013652.2	CCAGGGTTCTCAGCACCAAT	TCAGGAATACCACAGCTGGC
Ccl7	NM 013654	CGCTGCTTTCAGCATCCAAG	CTTGAAGATAACAGCTTCCCAGG
Cd274	NM 021893.2	TTCACAGCCTGCTGTCACTT	TAAGGTCCTCCTCTCCTGCC
Cd3d	NM 013487.1	ATTGCTGTTCTCCCCCAAGG	AACCATCCTTCCACCGTTCC
Cd8b1	NM 009858.2	CCTGACATGCAGCCTTACCA	CTCTCCTCCGCACACAGTAA
Cxcl1	NM 008176.1	CTGGGATTCACCTCA	CAGGGTCAAGGCAAG
Edn2	NM 007902.2	TGTGCTACCTTCTGCCATCG	TGAGATATCCCTCAGCTTTCGG
Gbp2	NM 010260.1	CCAAGAAGGCAGGGCAAACC	CCGTCACATAGTGCAGCTGGT
Gbp3	NM 018734.3	AGTAGTGTTCCCTGGGTTCTG	AAACCAACAATGGCCACCACC
Gbp6	NM 145545.2	CAGCAAGCCCAAGTTCACAC	GGCGAAGATCCACGAGTCAT
Gzmg	NM 010375.2	CTGGAGCAGAGGAGATCATCG	ATGTCATTGGTGCCATGCTTT
H2-Aa	NM 010378.2	TACCAATGAGGCTCCTCAAGC	CGTCTGCGACTGACTTGCTA
H2-Eb1	NM 010382.2	CGGTCGAGTGGAAAGCACAA	AAGTAGATGAACAGCCCCGC
Igtp	NM 018738	TCTCTCTCCTGACCGTCCTG	GTGCTCCTCGGCTTCTTTCT
Irgb10	XM 905096.3	GTGGTCCATGTCTGTGGAGG	ATTCGGCTCACTTGACAGCA
Irgm1	NM 008326.1	ATAACTCCTCTGGATCAGGGTTT	CTCAGGTTCTTCCTCCACTCT
S100a9	NM 009114.1	GGAGGACCTGGACACAAACC	TACTTCCCACAGCCTTTGCC
