# Supplementary material for: Different genotypes of Trypanosoma cruzi produce distinctive placental environment genetic response in chronic experimental infection
Source: PLoS Negl Trop Dis. 2017 Mar 8;11(3):e0005436. doi: 10.1371/journal.pntd.0005436 (PMC5358786; doi:10.1371/journal.pntd.0005436)
Supplement: S2 Table — (PDF) [file pntd.0005436.s002.pdf]

S2 Table. Fold Change and adjusted p-values from Microarray analysis.

Refseq_NM	GeneSymbol	Gene_ID	K98/Control.FC	K98/Control.adj p-value
NM_001081273.1	1600015I10Rik	69761	-2,611623	1,45E-07
NM_025929.2	2010109I03Rik	67038	-2,735597	7,60E-17
XM_130053.2	2010317E24Rik		-1,929421	0,018582956
XM_916650.3	2310043J07Rik	69665	2,321376	0
NM_178098.2	4930486L24Rik	214639	-1,958369	8,25E-05
XM_001478116.1	4933400N17Rik	619289	-2,146601	0,003252443
XM_127434	9030624O13Rik		-2,635933	0,000916733
NM_009338.3	Acat2	110460	-1,894998	0,004211564
NM_009022.2	Aldh1a2	19378	-2,997067	9,81E-05
NM_009022.3	Aldh1a2	19378	-2,224799	0,019485098
NM_053080.2	Aldh1a3	56847	1,73214	0,008345209
NM_020036.4	Calm4	80796	1,678101	0,004336831
NM_013652.2	Ccl4	20303	2,507951	0,000495927
NM_013653.2	Ccl5	20304	3,114661	9,81E-05
NM_019950.2	Chst5	56773	-1,950646	0,019740092
NM_007728.2	Coch	12810	2,893535	7,70E-06
NM_139229.3	Cog8	97484	-2,139679	0,044956745
NM_009935.1	Col6a3	12835	-1,832999	0,036009232
NM_013496.2	Crabp1	12903	1,798079	0,036009232
NM_008411.3	Cuzd1	16433	-2,575716	0,019485098
NM_008176.1	Cxcl1	14825	3,007446	0,000342845
NM_019568.2	Cxcl14	57266	-1,753257	0,021847105
NM_001029987.1	Doxl2	243376	-1,882367	4,18E-05
NM_001029987.1	Doxl2	243376	-1,821769	0,011006505
NM_007895.2	Ear2	13587	-1,771954	0,004604266
NM_007895.2	Ear2	13587	-1,735742	0,003199546
NM_007895.2	Ear2	13587	-1,659634	0,007067226
NM_017388.1	Ear3	53876	-1,645913	0,001908503
NM_001017422.1	Ear4	53877	-1,797612	0,00032144
NM_007902.2	Edn2	13615	3,620466	1,52E-07
NM_010332.2	Ednra	13617	-1,840176	0,006653658
NM_201256.2	Eif4ebp3	108112	-1,634598	0,044325322
AK089567	F830002E14Rik		2,26917	0,00804542
NM_010188.4	Fcgr3	14131	-1,777415	0,014022856
NM_008009.2	Fgfbp1		1,967485	0,000618517
NM_028263.1	Fgfbp3	72514	-2,452469	0,000820362
NM_010216.1	Figf	14205	-2,460956	0,002882989
NM_008191.1	Guca2b	14916	-3,102059	3,05E-07

NM_010372.1	Gzmd	14941	-3,435688	0,000167553
NM_010375.2	Gzmg	14944	-3,139537	0,002700925
NM_008221.2	Hbb-y	15135	2,89731	0,000147874
NM_008221.2	Hbb-y	15135	2,929904	0,000391962
NM_008309.3	Htr1d	15552	-2,622771	4,30E-06
NM_008330.1	Ifi47	15953	2,074102	0,019560975
XM_001472091.1	Igh-VJ558	16061	-1,658924	0,045320769
NM_018738.3	Igtp	16145	2,603193	0,000427117
NM_009349.1	Inmt	21743	-3,584352	9,82E-16
NM_009349.3	Inmt	21743	-2,838754	2,38E-14
NM_008407	Itih3		-1,766531	0,000430171
NM_001038607.1	Kcnh1	16510	-1,986574	0,003895833
NM_008940.2	Klk8	259277	2,017004	0,000304142
NM_010662.1	Krt13	16663	1,903555	3,11E-07
NM_008469.1	Krt15	16665	1,739707	0,016126698
XM_001478360.1	LOC100042514	100042514	-2,742379	7,65E-08
XM_977361.1	LOC665506	665506	2,17558	0,017583162
NM_008509.2	Lpl	16956	-1,928756	0,000683859
NM_177303.4	Lrrn4	320974	-2,340159	0,002381771
NM_023463.3	Ly6g6c	68468	-1,737138	0,026206525
NM_133743.1	Lypd3	72434	1,655566	0,006448314
NM_010762.4	Mal	17153	1,783347	4,14E-05
NM_010762.4	Mal	17153	2,00013	1,22E-08
NM_015776.2	Mfap5	50530	1,966989	0,021090507
XM_911929.3	Muc16	73732	2,334421	2,87E-12
NM_175024.3	Nrn1l	234700	-2,072886	0,008694156
NM_016968.4	Olig1	50914	-2,526019	0,000576724
NM_145932.3	Osta	106407	-2,077838	0,014649926
NM_178933.2	Ostb	330962	-3,060914	1,11E-11
NM_133200.3	P2ry14	140795	-2,867454	0,00027545
NM_133200.3	P2ry14	140795	-2,56593	0,001414769
NM_153565.1	Pcsk9	100102	-1,894878	8,85E-05
NM_001024145.1	Pla2g4f	271844	1,535122	0,011680044
NM_023785.2	Ppbp	57349	1,695886	0,03436742
NM_023785.1	Ppbp	57349	1,717149	0,000388842
NM_009475.1	Prap1	22264	-2,149491	0,002882989
NM_019991.1	Prl2a1	56635	1,614383	0,040654901
NM_026206.2	Prl7c1	67505	1,73033	0,008144296
NM_023746.3	Prlpl	28078	-2,249502	0,003199546
XM_127254.1	Prlpn		1,855823	0,045320769
XM_001475793.1	Rarres1	109222	-1,718788	0,004217195

NM_181596.3	Retnlg	245195	2,426414	0,015871306
NM_009062.3	Rgs4	19736	-1,940898	0,009952353
NM_009114.1	S100a9	20202	2,462287	0,000675813
NM_011315.3	Saa3	20210	2,330923	0,00013952
NM_011681.2	Scgb1a1	22287	-2,534808	3,64E-05
NM_172953.1	Serpina5	268591	-2,037588	0,026299916
NM_009776.1	Serping1	12258	-1,631042	0,046218629
NM_009160.1	Sftpd	20390	-1,585917	5,86E-07
NM_194333.3	Slc23a3	22626	-1,56289	0,038858877
NM_011773	Slc30a3		-2,203665	0,000728395
NM_175316.3	Slco2b1	101488	-2,01862	5,73E-06
NM_009234.3	Sox11		-2,504781	4,14E-05
NM_009234.5	Sox11	20666	-2,232837	0,000607415
NM_009181.1	St8sia2	20450	-1,738575	0,019560975
NM_011638.3	Tfrc	22042	-3,69901	2,30E-17
NM_011638.3	Tfrc	22042	-3,672227	2,18E-18
NM_011581.1	Thbs2	21826	-1,691707	0,021253846
NM_009414.2	Tph1	21990	-3,91624	7,17E-10
NM_009504.3	Vdr	22337	-2,29761	0,027711498
NM_178909.4	Wdr92	103784	-1,569016	0,014599395
NM_153541.3	Zbtb8b	215627	-2,075331	0,01614109

Refseq_NM	GeneSymbol	Gene_ID	VD/Control.FC	VD/Control.adj p-value
	0610037M15Rik		2,348159	0,048532657
NM_134054.2	1110002B05Rik	104725	-2,034264	0,000119373
NM_024244	1200015N20Rik		-2,088994	0,00983389
XM_356401.5	1700123J19Rik	73614	2,272474	0,026411758
NM_027237.1	2010003K11Rik	69861	-1,957971	0,027202375
NM_025929.2	2010109I03Rik	67038	-1,75882	6,10791E-06
NM_025501.1	2310007F04Rik	66344	2,46359	0,000155467
XM_916650.3	2310043J07Rik	69665	2,131408	0,001265412
NM_178098.2	4930486L24Rik	214639	-2,148311	1,98112E-05
	A130010C12Rik		1,560261	0,008966394
AK037444	A130019H11Rik		2,194289	0,002239151
XR_001572.1	A330102K04Rik	328563	2,089129	0,040040477
NM_001004174.1	AA467197	433470	2,17647	4,90467E-07
NM_009338.3	Acat2	110460	-2,027805	0,000703496
NM_172723.4	Adap1	231821	-2,0523	0,002564815
NM_001033207.2	AI451557	102084	2,509093	9,96139E-05
XM_980696.2	AI607873	226691	2,11253	0,007947118
NM_134066.2	Akr1c18	105349	-2,359189	1,27316E-06
NM_009022.2	Aldh1a2	19378	-3,659045	2,94704E-10

NM_009022.3	Aldh1a2	19378	-2,517509	0,00011127
NM_008486.1	Anpep	16790	1,891643	0,000359213
NM_173743.3	Apol9b	71898	3,33526	1,03178E-08
NM_009807.2	Casp1	12362	2,865144	2,9158E-07
NM_011337.2	Ccl3	20302	3,336458	1,11931E-08
NM_013652.2	Ccl4	20303	4,44517	0
NM_013652.2	Ccl4	20303	10,596221	0
NM_013653.2	Ccl5	20304	8,566627	0
NM_013654.2	Ccl7	20306	3,196578	1,11931E-08
NM_013654	Ccl7		3,635001	1,58636E-11
NM_009917.2	Ccr5	12774	2,085242	0,001797561
NM_021893.2	Cd274	60533	5,402823	0
NM_013487.1	Cd3d		2,862941	1,1444E-05
NM_170702.2	Cd40	21939	2,010803	0,001172531
NM_013706.1	Cd52	23833	2,393968	0,000255617
NM_009852.3	Cd6	12511	1,936829	0,043906642
NM_009852.3	Cd6	12511	1,980373	0,049141036
NM_001042605.1	Cd74	16149	5,741372	0
NM_010545.3	Cd74	16149	5,830354	0
NM_009858.2	Cd8b1	12526	3,47692	3,43755E-07
NM_009892.1	Chi3l3	12655	2,541478	1,68679E-05
NM_009892	Chi3l3		2,653323	9,31698E-08
NM_145126.1	Chi3l4	104183	2,522602	1,08497E-05
NM_021386.3	Cldn10	58187	-2,171752	9,02599E-06
NM_023878.2	Cldn10	58187	-2,017496	0,004117319
NM_010819.3	Clec4d	17474	2,106585	0,008875608
NM_007730.2	Col12a1	12816	-1,815221	0,046222989
NM_009935.1	Col6a3	12835	-1,770963	0,005289302
NM_013494.2	Cpe		-1,651363	0,000330979
NM_013496.2	Crabp1	12903	2,478582	4,40501E-06
NM_011804.2	Creg1	433375	-1,952592	0,000155467
NM_029720.1	Creld2	76737	-1,624876	0,027980939
NM_029720.2	Creld2	76737	-1,589911	0,0431081
NM_033616.3	Csprs	114564	2,3699	0,00011127
NM_010217	Ctgf		1,628157	0,001223264
NM_010217.1	Ctgf	14219	1,69414	8,83433E-06
NM_026906.2	Cts3	117066	-1,618916	0,025018077
NM_022326.3	Ctsm	64139	-1,554455	0,028901924
NM_019568.2	Cxcl14	57266	-2,047506	3,41646E-06
NM_019568.1	Cxcl14		-1,767754	0,001902962
NM_008599.3	Cxcl9	17329	17,027535	0

NM_199015.2	D14Ertd668e	219132	2,178118	0,019497374
NM_199015.1	D14Ertd668e	219132	2,22515	0,002923617
NM_007833.4	Dcn	13179	-1,782712	0,01010367
AK008179	Dlm1-pending		2,103932	0,049141036
NM_007870.2	Dnase1l3		1,791991	0,009707565
NM_001033366.1	Dpcr1	268949	1,591216	0,014213227
NM_176913.3	Dpep2	319446	-2,000822	0,002377683
NM_176913	Dpep2		-1,923445	0,020855798
NM_001013371.2	Dtx3l	209200	2,023238	0,000270388
NM_053112.1	Ear10	93725	-2,061652	3,56833E-05
NM_001012766.1	Ear12	503845	-2,373598	3,4833E-07
NM_007895.2	Ear2	13587	-2,130879	1,10068E-06
NM_007895.2	Ear2	13587	-1,816165	3,72468E-05
NM_007895.2	Ear2	13587	-1,801217	4,28831E-06
NM_017388.1	Ear3	53876	-1,975093	0,000158955
NM_017388.1	Ear3	53876	-1,931789	2,19602E-06
NM_017388.1	Ear3	53876	-1,81001	0,000175942
NM_001017422.1	Ear4	53877	-2,034687	1,3656E-06
NM_010336.1	Edg2	14745	-1,749573	0,00312003
NM_007902.2	Edn2	13615	4,938977	0
NM_010332.2	Ednra	13617	-1,979837	0,000422142
NM_001033767.2	EG240327	240327	4,775423	0
NM_201256.2	Eif4ebp3	108112	-1,700047	0,004529009
AK089567	F830002E14Rik		2,207336	6,28316E-06
NM_024244.3	Fam13c	71721	-2,34364	0,000307342
NM_145946.2	Fanci	208836	-1,990361	0,04681751
NM_175401.3	Fbxw17	109082	1,790252	0,006419478
NM_144559.1	Fcgr4	246256	3,163253	3,18136E-09
NM_028263.1	Fgfbp3	72514	-2,668611	2,59682E-06
NM_008013.3	Fgl2	14190	-1,790955	0,026697587
NM_010216.1	Figf	14205	-2,253501	0,011178866
NM_010231.2	Fmo1	14261	-1,772374	0,002691882
NM_008039.2	Fpr2	14289	4,927953	0
NM_010260.1	Gbp2	14469	7,551665	0
NM_018734.2	Gbp3	55932	3,887224	0
NM_018734.3	Gbp3	55932	4,279849	0
NM_145545.2	Gbp6	229900	2,019011	0,003524279
NM_145545.2	Gbp6	229900	2,12188	0,000144083
AK089366	Gp49a		2,046424	0,014213227
NM_026960.1	Gsdmdc1	69146	1,910673	0,002239151
NM_026960.3	Gsdmdc1	69146	2,060093	0,000344259

NM_008191.1	Guca2b	14916	-2,978411	7,55722E-08
NM_172810.2	Gucy1b2	239134	-1,731048	0,015405293
NM_001039160.2	Gvin1	74558	2,764862	3,46923E-08
NM_010372.1	Gzmd	14941	-2,154716	2,29263E-08
NM_010372.2	Gzmd	14941	-2,14523	6,07786E-05
NM_010373.2	Gzme		-1,946759	0,000317088
NM_010373.3	Gzme	14942	-1,844107	0,000843073
NM_010375.2	Gzmg	14944	-2,162604	3,19643E-07
NM_010378.2	H2-Aa	14960	3,760857	2,70399E-10
NM_207105.2	H2-Ab1	14961	2,539023	2,40167E-06
NM_207105.1	H2-Ab1	14961	2,86936	1,58733E-08
NM_207105.2	H2-Ab1	14961	3,673354	0
NM_010387.2	H2-DMb1	14999	2,53715	0,000173096
NM_010382.2	H2-Eb1	14969	4,737583	0
NM_023124.2	H2-Q8	15019	2,411084	0,023981902
NM_023124	H2-Q8		2,876567	0,000103536
NM_010395.5	H2-T10	15024	2,230112	0,017421696
NM_010395.2	H2-T10		2,438331	0,000973075
NM_001033245.3	Hk3	212032	2,608705	2,5444E-05
NM_008278	Hpgd		1,873258	0,000179459
NM_010493.2	Icam1	15894	2,356811	3,51523E-06
NM_008330.1	Ifi47	15953	3,82703	0
NM_008332.2	Ifit2	15958	4,432878	0
NM_008332.2	Ifit2	15958	5,411918	0
NM_010501.1	Ifit3	15959	2,657895	1,26627E-08
NM_025378.2	Ifitm3	66141	1,681806	0,008939025
NM_018738.3	Igtp	16145	7,267965	0
NM_019440.2	Iigp2	54396	3,577034	0
NM_008361	Il1b		2,46562	6,07786E-05
NM_010555.4	Il1r2	16178	-1,664148	0,011331633
NM_031167.3	Il1rn	16181	2,259257	0,004117319
NM_008324.1	Indo	15930	9,031017	0
NM_009349.1	Inmt	21743	-3,739503	1,80566E-18
NM_009349.3	Inmt	21743	-3,056483	5,73643E-17
NM_008390.1	Irf1	16362	1,928154	0,006812138
NM_008390.1	Irf1	16362	3,387584	0
NM_008390.1	Irf1	16362	3,885324	0
NM_016850.2	Irf7	54123	2,173659	4,87898E-05
XM_905897.2	Irg1	16365	6,513286	0
XM_905096.3	Irgb10	631323	3,396839	8,18382E-12
NM_008326.1	Irgm1	15944	3,713168	0

NM_008407	Itih3		-2,306407	1,07753E-12
NM_008407.1	Itih3	16426	-2,233902	6,42906E-08
NM_001038607.1	Kcnh1	16510	-1,982909	0,031707053
NM_010645.2	Klk1b1	16623	-2,529474	0,02129467
NM_010645.2	Klk1b1	16623	-2,512734	0,027980939
NM_010642.1	Klk1b21	16616	-2,349454	0,000239669
NM_020268.1	Klk1b27	16619	-2,518375	4,78817E-05
NM_020268.3	Klk1b27	16619	-2,077949	0,002118019
NM_010915.1	Klk1b4	18048	-2,141255	0,002600578
NM_008456.2	Klk1b5	16622	-2,666889	1,15415E-06
NM_008940.2	Klk8	259277	1,654865	0,001061445
NM_010662.1	Krt13	16663	1,823921	0,000521642
NM_008469.1	Krt15	16665	1,934742	5,73081E-05
NM_008470.1	Krt16	16666	2,109492	5,3972E-10
NM_001033131.1	Krtdap	64661	1,710166	4,12626E-06
NM_033175.2	Lce3c	94060	3,247628	1,99811E-08
NM_008491.1	Lcn2	16819	2,467281	5,22827E-06
XM_283647	Lgr6		-2,278667	0,001384357
NM_001033409.3	Lgr6	329252	-2,091419	0,002564815
NM_144799.1	Lmcd1	30937	2,057427	0,01246225
NM_144799.1	Lmcd1	30937	2,101829	0,006665915
XM_001471686.1	LOC100038882	100038882	2,160817	0,002520024
XM_001478360.1	LOC100042514	100042514	-2,456952	1,1847E-06
XM_001471649.1	LOC100044190	100044190	2,855246	4,19215E-08
XM_001471649.1	LOC100044190	100044190	2,981027	3,484E-08
XM_001472240.1	LOC100044430	100044430	2,598921	2,7942E-05
XM_001472253.1	LOC100044439	100044439	1,875117	0,024592777
XM_001480891.1	LOC100048556	100048556	2,375209	0,000169128
XM_128064.4	LOC223672		3,684725	1,50816E-11
XM_354621.1	LOC380706		2,015018	0,000281467
XM_914287.2	LOC638301	638301	3,119567	1,26627E-08
XM_918601.3	LOC641240	641240	3,646283	7,73416E-13
XM_977361.1	LOC665506	665506	4,541653	1,71661E-12
XM_001002943.1	LOC677375	677375	3,79777	0
NM_010728.1	Lox	16948	-2,270429	0,000143864
NM_008524.1	Lum	17022	-1,813745	0,034564664
NM_010738.2	Ly6a	110454	2,035983	4,85484E-10
NM_008529	Ly6e		1,799091	0,00399098
NM_133743.1	Lypd3	72434	2,039031	1,01527E-06
NM_010762.4	Mal	17153	1,638019	0,001061048
NM_015776.2	Mfap5	50530	2,177766	1,68679E-05

NM_029005.1	Mlkl	74568	2,115738	0,032908728
NM_026835.2	Ms4a6d	68774	2,170688	0,01694203
NM_026835.2	Ms4a6d	68774	2,604744	1,87771E-05
NM_010827.2	Msc	17681	-2,611217	0,002377683
XM_911929.3	Muc16	73732	2,127688	1,78713E-07
NM_024253.4	Nkg7	72310	2,724928	0,000359213
NM_175024.3	Nrn1l	234700	-1,752167	0,014906018
NM_145227.1	Oas2	246728	2,097103	0,015123345
NM_011854.1	Oasl2	23962	1,784203	0,001490235
NM_011854.2	Oasl2	23962	1,87565	0,008909156
NM_016968.4	Olig1	50914	-2,54625	3,39042E-05
NM_145932.3	Osta	106407	-2,06518	0,005548737
NM_178933.2	Ostb	330962	-2,874687	6,04019E-16
NM_133200.3	P2ry14	140795	-2,198109	0,009059792
NM_133200.3	P2ry14	140795	-2,026772	0,014759706
NM_001039530.1	Parp14	547253	2,149176	3,16063E-06
NM_153565.1	Pcsk9	100102	-1,818257	1,92077E-05
NM_016798.2	Pdlim3	53318	-2,480904	4,6635E-06
NM_016798.3	Pdlim3	53318	-2,213977	6,14943E-05
NM_008872	Plat		-1,821539	0,003541067
NM_011961	Plod2		-1,748948	0,036753009
NM_054077.3	Prelp	116847	1,551378	0,031503604
NM_145984.2	Prepl	213760	-1,744227	0,025213351
NM_013766.1	Prl3c1	27372	-1,603931	0,015405293
NM_023332.3	Prl8a9	67310	-1,897336	0,006365371
NM_023746.3	Prlpl	28078	-2,17444	0,003103995
XM_127254.1	Prlpn		1,741836	0,018799137
NM_011963.1	Psg18	26438	-2,095164	3,4833E-07
NM_011963.1	Psg18	26438	-2,050132	0,000218681
NM_011963.1	Psg18	26438	-2,004059	3,02319E-06
NM_011964.1	Psg19	26439	-1,83968	0,031441401
NM_013640.1	Psmb10	19171	2,138898	6,81151E-05
NM_013640.2	Psmb10	19171	2,15573	0,000319796
NM_013585.2	Psmb9	16912	4,34972	0
NM_027455.1	Qpct	70536	-1,577953	0,023630261
NM_001040005.2	Rnf213	629974	1,952149	0,000930941
NM_021384.3	Rsad2	58185	1,666692	0,01074902
NM_011315.3	Saa3	20210	4,22347	0
NM_011681.2	Scgb1a1	22287	-2,450301	1,01705E-06
NM_009251.1	Serpina3g	20715	6,845982	0
NM_172953.1	Serpina5	268591	-1,7737	0,025018077

NM_009255.2	Serpine2	20720	-1,871552	0,01074902
AK045954	Serpine2		-1,665302	0,005548737
NM_019982.2	Sez6l	56747	1,820519	0,012965503
NM_016687.2	Sfrp4	20379	-2,090053	0,002218766
XM_129596.2	Slamf8		3,720887	6,84685E-10
NM_023044.1	Slc15a3	65221	2,301212	0,002218766
NM_172659.2	Slc2a6	227659	3,505101	7,08333E-09
NM_175316.3	Slco2b1	101488	-1,767309	7,71435E-11
NM_011407.1	Slfn1	20555	2,070726	0,006454042
NM_007707.2	Socs3	12702	1,891831	0,001794276
NM_009263.1	Spp1	20750	-1,730041	0,000103536
NM_011470.1	Sprr2d	20758	2,087397	2,8724E-05
NM_011157.2	Srgn	19073	-1,817514	0,006783413
NM_009283.3	Stat1	20846	2,37933	1,48476E-05
NM_001082543.1	Stfa1	20861	1,503125	0,000175942
NM_026716.2	Sycn	68416	-1,676614	0,018799137
NM_013683.1	Tap1	21354	3,019332	3,1588E-08
NM_011530.2	Tap2	21355	2,276822	2,8932E-05
NM_011638.3	Tfrc	22042	-2,703625	2,57855E-13
NM_011638.3	Tfrc	22042	-2,683345	3,81623E-12
NM_009369.1	Tgfbi	21810	1,863534	0,004922039
NM_011581.1	Thbs2	21826	-1,850529	0,047697241
NM_011905.2	Tlr2	24088	2,156339	0,009777016
NM_009414.2	Tph1	21990	-3,696545	3,83479E-08
NM_009277.3	Trim21	20821	2,023357	0,007224353
NM_020557.3	Tyki		2,128645	0,001510244
NM_023137.2	Ubd	24108	8,26174	0
NM_023738.4	Ube1l	74153	2,485984	1,64874E-07
NM_009504.2	Vdr	22337	-2,093386	0,009212267
NM_009504.3	Vdr	22337	-2,027089	0,009997111
NM_009524.2	Wnt5a	22418	-1,796122	0,01050335
NM_153541.3	Zbtb8b	215627	-1,820433	0,037018039
