# Supplementary material for: Different genotypes of Trypanosoma cruzi produce distinctive placental environment genetic response in chronic experimental infection
Source: PLoS Negl Trop Dis. 2017 Mar 8;11(3):e0005436. doi: 10.1371/journal.pntd.0005436 (PMC5358786; doi:10.1371/journal.pntd.0005436)
Supplement: S3 Table — (PDF) [file pntd.0005436.s003.pdf]

S3 Table. GeneMANIA Results for K98 group.

GO ID	DESCRIPTION	q-VALUE	OCCURRENCES IN SAMPLE	OCCURRENCES IN GENOME
GO:0050900	LEUKOCYTE MIGRATION	2.71E-09	12	199
GO:0060326	CELL CHEMOTAXIS	3.38E-10	11	174
GO:0030595	LEUKOCYTE CHEMOTAXIS	3.38E-10	10	126
GO:0097529	MYELOID LEUKOCYTE MIGRATION	8.36E-09	9	107
GO:0071621	GRANULOCYTE CHEMOTAXIS	1.57E-10	7	71
GO:0097530	GRANULOCYTE MIGRATION	2.11E-12	7	76
GO:0002687	POSITIVE REGULATION OF LEUKOCYTE MIGRATION	2.17E-12	7	78
GO:0009636	RESPONSE TO TOXIC SUBSTANCE	2.60E-11	7	83
GO:0032103	POSITIVE REGULATION OF RESPONSE TO EXTERNAL STIMULUS	2.60E-11	9	172
GO:0002690	POSITIVE REGULATION OF LEUKOCYTE CHEMOTAXIS	2.98E-11	6	52
GO:0030593	NEUTROPHIL CHEMOTAXIS	5.26E-11	6	58
GO:1990266	NEUTROPHIL MIGRATION	5.35E-11	6	59
GO:0002685	REGULATION OF LEUKOCYTE MIGRATION	8.43E-11	7	105
GO:0002688	REGULATION OF LEUKOCYTE CHEMOTAXIS	8.43E-11	6	65
GO:0050921	POSITIVE REGULATION OF CHEMOTAXIS	0.0046	6	88
GO:0005501	RETINOID BINDING	0.0091	4	27
GO:0048520	POSITIVE REGULATION OF BEHAVIOR	0.0114	6	105
GO:0008009	CHEMOKINE ACTIVITY	0.0119	4	30
GO:0019840	ISOPRENOID BINDING	0.0119	4	30
GO:0050729	POSITIVE REGULATION OF INFLAMMATORY RESPONSE	0.0132	5	65
GO:0002523	LEUKOCYTE MIGRATION INVOLVED IN INFLAMMATORY RESPONSE	0.0155	3	11
GO:0004252	SERINE-TYPE ENDOPEPTIDASE ACTIVITY	0.0161	5	69
GO:0050920	REGULATION OF CHEMOTAXIS	0.0164	6	118
GO:0005125	CYTOKINE ACTIVITY	0.0181	6	121
GO:0002526	ACUTE INFLAMMATORY RESPONSE	0.0213	5	75
GO:0001972	RETINOIC ACID BINDING	0.0215	3	13
GO:0072330	MONOCARBOXYLIC ACID BIOSYNTHETIC PROCESS	0.0275	6	133
GO:0042379	CHEMOKINE RECEPTOR BINDING	0.0295	4	42
GO:0015721	BILE ACID AND BILE SALT TRANSPORT	0.0295	3	15
GO:0050727	REGULATION OF INFLAMMATORY RESPONSE	0.0295	7	202
GO:0014821	PHASIC SMOOTH MUSCLE CONTRACTION	0.0350	3	16
GO:0008236	SERINE-TYPE PEPTIDASE ACTIVITY	0.0410	5	91
GO:0014829	VASCULAR SMOOTH MUSCLE CONTRACTION	0.0410	3	17
GO:0007205	PROTEIN KINASE C-ACTIVATING G-PROTEIN COUPLED RECEPTOR SIGNALING PATHWAY	0.0462	3	18
GO:0002673	REGULATION OF ACUTE INFLAMMATORY RESPONSE	0.0462	4	49
GO:0017171	SERINE HYDROLASE ACTIVITY	0.0462	5	95
GO:0046457	PROSTANOID BIOSYNTHETIC PROCESS	0.0556	3	20

GO:0033293	MONOCARBOXYLIC ACID BINDING	0.0556	4	53
GO:0014032	NEURAL CREST CELL DEVELOPMENT	0.0556	4	53
GO:0001516	PROSTAGLANDIN BIOSYNTHETIC PROCESS	0.0556	3	20
GO:0050795	REGULATION OF BEHAVIOR	0.0707	6	170
GO:0008015	BLOOD CIRCULATION	0.0707	7	245
GO:0014033	NEURAL CREST CELL DIFFERENTIATION	0.0736	4	58
GO:0046677	RESPONSE TO ANTIBIOTIC	0.0762	3	23
GO:0002675	POSITIVE REGULATION OF ACUTE INFLAMMATORY RESPONSE	0.0849	3	24
