# Supplementary material for: Different genotypes of Trypanosoma cruzi produce distinctive placental environment genetic response in chronic experimental infection
Source: PLoS Negl Trop Dis. 2017 Mar 8;11(3):e0005436. doi: 10.1371/journal.pntd.0005436 (PMC5358786; doi:10.1371/journal.pntd.0005436)
Supplement: S4 Table — (PDF) [file pntd.0005436.s004.pdf]

S4 Table. GeneMANIA Results for VD group.

GO ID	DESCRIPTION	Q-VALUE	OCCURRENCES IN SAMPLE	OCCURRENCES IN GENOME
GO:0034341	RESPONSE TO INTERFERON-GAMMA	2.40E-02	15	54
GO:0045087	INNATE IMMUNE RESPONSE	4.90E-02	24	266
GO:0019882	ANTIGEN PROCESSING AND PRESENTATION	1.85E-04	16	92
GO:0035456	RESPONSE TO INTERFERON-BETA	3.17E-03	11	28
GO:0035458	CELLULAR RESPONSE TO INTERFERON-BETA	1.59E-05	10	23
GO:0019884	ANTIGEN PROCESSING AND PRESENTATION OF EXOGENOUS ANTIGEN	9.46E-04	10	27
GO:0048002	ANTIGEN PROCESSING AND PRESENTATION OF PEPTIDE ANTIGEN	1.62E-06	12	53
GO:0002478	ANTIGEN PROCESSING AND PRESENTATION OF EXOGENOUS PEPTIDE ANTIGEN	2.02E-05	9	21
GO:0043245	EXTRAORGANISMAL SPACE	2.02E-05	8	14
GO:0044216	OTHER ORGANISM CELL	2.02E-05	8	14
GO:0044215	OTHER ORGANISM	2.02E-05	8	14
GO:0044217	OTHER ORGANISM PART	2.02E-05	8	14
GO:0018995	HOST	2.02E-05	8	14
GO:0043657	HOST CELL	2.02E-05	8	14
GO:0009617	RESPONSE TO BACTERIUM	4.17E-06	21	287
GO:0009897	EXTERNAL SIDE OF PLASMA MEMBRANE	8.33E-05	20	265
GO:0002687	POSITIVE REGULATION OF LEUKOCYTE MIGRATION	8.87E-06	12	78
GO:0042611	MHC PROTEIN COMPLEX	1.01E-08	7	13
GO:0071346	CELLULAR RESPONSE TO INTERFERON-GAMMA	3.50E-07	9	36
GO:0002690	POSITIVE REGULATION OF LEUKOCYTE CHEMOTAXIS	4.36E-07	10	52
GO:0098542	DEFENSE RESPONSE TO OTHER ORGANISM	4.79E-07	17	229
GO:0044403	SYMBIOSIS, ENCOMPASSING MUTUALISM THROUGH PARASITISM	6.23E-07	15	172
GO:0044419	INTERSPECIES INTERACTION BETWEEN ORGANISMS	6.23E-07	15	172
GO:0032103	POSITIVE REGULATION OF RESPONSE TO EXTERNAL STIMULUS	6.23E-07	15	172
GO:0033655	HOST CELL CYTOPLASM PART	8.69E-07	6	10
GO:0033646	HOST INTRACELLULAR PART	8.69E-07	6	10
GO:0001819	POSITIVE REGULATION OF CYTOKINE PRODUCTION	8.69E-07	16	208
GO:0043656	INTRACELLULAR REGION OF HOST	8.69E-07	6	10
GO:0030430	HOST CELL CYTOPLASM	8.69E-07	6	10
GO:0002237	RESPONSE TO MOLECULE OF BACTERIAL ORIGIN	9.50E-07	15	180
GO:0033643	HOST CELL PART	1.67E-09	6	11
GO:0048520	POSITIVE REGULATION OF BEHAVIOR	1.67E-09	12	105
GO:0002685	REGULATION OF LEUKOCYTE MIGRATION	1.67E-09	12	105
GO:0002688	REGULATION OF LEUKOCYTE CHEMOTAXIS	2.61E-08	10	65
GO:0050921	POSITIVE REGULATION OF CHEMOTAXIS	3.33E-08	11	88

GO:0060326	CELL CHEMOTAXIS	5.30E-08	14	174
GO:0009615	RESPONSE TO VIRUS	5.53E-08	13	145
GO:0042832	DEFENSE RESPONSE TO PROTOZOAN	8.83E-08	7	24
GO:0019886	ANTIGEN PROCESSING AND PRESENTATION OF EXOGENOUS PEPTIDE ANTIGEN VIA MHC CLASS II	8.96E-08	6	14
GO:0031349	POSITIVE REGULATION OF DEFENSE RESPONSE	1.12E-10	14	186
GO:0030595	LEUKOCYTE CHEMOTAXIS	1.12E-10	12	126
GO:0071219	CELLULAR RESPONSE TO MOLECULE OF BACTERIAL ORIGIN	1.24E-10	11	101
GO:0001562	RESPONSE TO PROTOZOAN	1.96E-08	7	27
GO:0002460	ADAPTIVE IMMUNE RESPONSE BASED ON SOMATIC RECOMBINATION OF IMMUNE RECEPTORS BUILT FROM IMMUNOGLOBULIN SUPERFAMILY DOMAINS	2.43E-09	13	166
GO:0050900	LEUKOCYTE MIGRATION	2.43E-09	14	199
GO:0002495	ANTIGEN PROCESSING AND PRESENTATION OF PEPTIDE ANTIGEN VIA MHC CLASS II	3.05E-09	6	17
GO:0002819	REGULATION OF ADAPTIVE IMMUNE RESPONSE	3.05E-09	11	111
GO:0071216	CELLULAR RESPONSE TO BIOTIC STIMULUS	3.61E-09	11	113
GO:0002504	ANTIGEN PROCESSING AND PRESENTATION OF PEPTIDE OR POLYSACCHARIDE ANTIGEN VIA MHC CLASS II	4.27E-09	6	18
GO:0050920	REGULATION OF CHEMOTAXIS	5.49E-09	11	118
GO:0002449	LYMPHOCYTE MEDIATED IMMUNITY	5.66E-09	12	148
GO:0003823	ANTIGEN BINDING	6.73E-09	9	70
GO:0045088	REGULATION OF INNATE IMMUNE RESPONSE	8.51E-09	12	154
GO:0042605	PEPTIDE ANTIGEN BINDING	8.94E-09	7	34
GO:0051701	INTERACTION WITH HOST	9.54E-09	8	52
GO:0019221	CYTOKINE-MEDIATED SIGNALING PATHWAY	9.88E-09	14	226
GO:0032496	RESPONSE TO LIPOPOLYSACCHARIDE	1.30E-11	12	161
GO:0050795	REGULATION OF BEHAVIOR	2.34E-10	12	170
GO:0002250	ADAPTIVE IMMUNE RESPONSE	2.55E-10	13	207
GO:0051825	ADHESION TO OTHER ORGANISM INVOLVED IN SYMBIOTIC INTERACTION	2.78E-10	5	13
GO:0042098	T CELL PROLIFERATION	3.57E-11	11	144
GO:0042379	CHEMOKINE RECEPTOR BINDING	3.61E-10	7	42
GO:0051702	INTERACTION WITH SYMBIONT	3.61E-10	7	42
GO:0002821	POSITIVE REGULATION OF ADAPTIVE IMMUNE RESPONSE	4.39E-10	8	64
GO:0002699	POSITIVE REGULATION OF IMMUNE EFFECTOR PROCESS	4.50E-10	10	117
GO:0060337	TYPE I INTERFERON SIGNALING PATHWAY	5.80E-10	5	15
GO:0071222	CELLULAR RESPONSE TO LIPOPOLYSACCHARIDE	6.44E-10	9	93
GO:0071357	CELLULAR RESPONSE TO TYPE I INTERFERON	8.13E-10	5	16
GO:0002443	LEUKOCYTE MEDIATED IMMUNITY	8.31E-10	12	194
GO:0016032	VIRAL PROCESS	8.40E-10	10	126
GO:0008009	CHEMOKINE ACTIVITY	8.60E-10	6	30
GO:0046651	LYMPHOCYTE PROLIFERATION	8.67E-10	13	234
GO:0071621	GRANULOCYTE CHEMOTAXIS	8.75E-10	8	71

GO:0032943	MONONUCLEAR CELL PROLIFERATION	8.86E-10	13	235
GO:0050670	REGULATION OF LYMPHOCYTE PROLIFERATION	9.01E-10	11	161
GO:0032944	REGULATION OF MONONUCLEAR CELL PROLIFERATION	9.47E-10	11	162
GO:0002822	REGULATION OF ADAPTIVE IMMUNE RESPONSE BASED ON SOMATIC RECOMBINATION OF IMMUNE RECEPTORS BUILT FROM IMMUNOGLOBULIN SUPERFAMILY DOMAINS	1.13E-11	9	101
GO:0050868	NEGATIVE REGULATION OF T CELL ACTIVATION	1.13E-11	8	74
GO:0070661	LEUKOCYTE PROLIFERATION	1.22E-12	13	243
GO:0070663	REGULATION OF LEUKOCYTE PROLIFERATION	1.22E-12	11	167
GO:0097530	GRANULOCYTE MIGRATION	1.34E-12	8	76
GO:0044764	MULTI-ORGANISM CELLULAR PROCESS	1.46E-11	10	136
GO:0050778	POSITIVE REGULATION OF IMMUNE RESPONSE	1.56E-12	14	292
GO:0097529	MYELOID LEUKOCYTE MIGRATION	1.69E-12	9	107
GO:0034340	RESPONSE TO TYPE I INTERFERON	1.69E-12	5	19
GO:0002474	ANTIGEN PROCESSING AND PRESENTATION OF PEPTIDE ANTIGEN VIA MHC CLASS I	2.22E-12	6	36
GO:0060759	REGULATION OF RESPONSE TO CYTOKINE STIMULUS	2.28E-12	7	57
GO:0002683	NEGATIVE REGULATION OF IMMUNE SYSTEM PROCESS	2.38E-12	12	219
GO:0034121	REGULATION OF TOLL-LIKE RECEPTOR SIGNALING PATHWAY	2.55E-12	6	37
GO:0050708	REGULATION OF PROTEIN SECRETION	2.72E-12	10	147
GO:0002824	POSITIVE REGULATION OF ADAPTIVE IMMUNE RESPONSE BASED ON SOMATIC RECOMBINATION OF IMMUNE RECEPTORS BUILT FROM IMMUNOGLOBULIN SUPERFAMILY DOMAINS	2.77E-12	7	59
GO:0009306	PROTEIN SECRETION	3.06E-12	11	186
GO:0042129	REGULATION OF T CELL PROLIFERATION	3.06E-12	9	116
GO:0048247	LYMPHOCYTE CHEMOTAXIS	3.39E-12	5	22
GO:0071396	CELLULAR RESPONSE TO LIPID	4.25E-12	11	193
GO:0005125	CYTOKINE ACTIVITY	4.25E-12	9	121
GO:0050707	REGULATION OF CYTOKINE SECRETION	4.49E-12	8	91
GO:0050729	POSITIVE REGULATION OF INFLAMMATORY RESPONSE	5.00E-11	7	65
GO:0050866	NEGATIVE REGULATION OF CELL ACTIVATION	5.03E-11	9	124
GO:0030335	POSITIVE REGULATION OF CELL MIGRATION	5.29E-11	13	283
GO:0032606	TYPE I INTERFERON PRODUCTION	5.57E-11	6	43
GO:0042742	DEFENSE RESPONSE TO BACTERIUM	5.57E-11	9	126
GO:0042130	NEGATIVE REGULATION OF T CELL PROLIFERATION	6.33E-10	6	44
GO:2000147	POSITIVE REGULATION OF CELL MOTILITY	6.34E-10	13	289
GO:0050863	REGULATION OF T CELL ACTIVATION	6.34E-10	11	203
GO:0001660	FEVER GENERATION	6.69E-11	4	12
GO:0004252	SERINE-TYPE ENDOPEPTIDASE ACTIVITY	6.88E-11	7	69
GO:0031012	EXTRACELLULAR MATRIX	6.90E-11	13	292
GO:0051272	POSITIVE REGULATION OF CELLULAR COMPONENT MOVEMENT	8.23E-11	13	297
GO:0050867	POSITIVE REGULATION OF CELL ACTIVATION	8.39E-11	11	210

GO:0051250	NEGATIVE REGULATION OF LYMPHOCYTE ACTIVATION	8.63E-11	8	101
GO:0002697	REGULATION OF IMMUNE EFFECTOR PROCESS	0.0011	11	216
GO:0050663	CYTOKINE SECRETION	0.0012	8	106
GO:0032846	POSITIVE REGULATION OF HOMEOSTATIC PROCESS	0.0012	7	76
GO:0001959	REGULATION OF CYTOKINE-MEDIATED SIGNALING PATHWAY	0.0014	6	51
GO:0051817	MODIFICATION OF MORPHOLOGY OR PHYSIOLOGY OF OTHER ORGANISM INVOLVED IN SYMBIOTIC INTERACTION	0.0015	6	52
GO:1901623	REGULATION OF LYMPHOCYTE CHEMOTAXIS	0.0016	4	15
GO:0043903	REGULATION OF SYMBIOSIS, ENCOMPASSING MUTUALISM THROUGH PARASITISM	0.0020	8	114
GO:0050672	NEGATIVE REGULATION OF LYMPHOCYTE PROLIFERATION	0.0020	6	55
GO:0035821	MODIFICATION OF MORPHOLOGY OR PHYSIOLOGY OF OTHER ORGANISM	0.0020	6	55
GO:0032945	NEGATIVE REGULATION OF MONONUCLEAR CELL PROLIFERATION	0.0020	6	55
GO:0048020	CCR CHEMOKINE RECEPTOR BINDING	0.0020	4	16
GO:0050718	POSITIVE REGULATION OF INTERLEUKIN-1 BETA SECRETION	0.0020	4	16
GO:0050716	POSITIVE REGULATION OF INTERLEUKIN-1 SECRETION	0.0020	4	16
GO:0002711	POSITIVE REGULATION OF T CELL MEDIATED IMMUNITY	0.0021	5	33
GO:0005539	GLYCOSAMINOGLYCAN BINDING	0.0021	8	116
GO:0002695	NEGATIVE REGULATION OF LEUKOCYTE ACTIVATION	0.0022	8	117
GO:0051249	REGULATION OF LYMPHOCYTE ACTIVATION	0.0023	12	283
GO:0070664	NEGATIVE REGULATION OF LEUKOCYTE PROLIFERATION	0.0024	6	57
GO:0042277	PEPTIDE BINDING	0.0025	10	196
GO:0050706	REGULATION OF INTERLEUKIN-1 BETA SECRETION	0.0025	4	17
GO:0030593	NEUTROPHIL CHEMOTAXIS	0.0025	6	58
GO:1990266	NEUTROPHIL MIGRATION	0.0028	6	59
GO:0002696	POSITIVE REGULATION OF LEUKOCYTE ACTIVATION	0.0028	10	200
GO:0006953	ACUTE-PHASE RESPONSE	0.0030	4	18
GO:0001961	POSITIVE REGULATION OF CYTOKINE-MEDIATED SIGNALING PATHWAY	0.0030	4	18
GO:0050727	REGULATION OF INFLAMMATORY RESPONSE	0.0030	10	202
GO:0031649	HEAT GENERATION	0.0030	4	18
GO:0033218	AMIDE BINDING	0.0031	10	203
GO:0002224	TOLL-LIKE RECEPTOR SIGNALING PATHWAY	0.0032	6	61
GO:0008236	SERINE-TYPE PEPTIDASE ACTIVITY	0.0033	7	91
GO:0032479	REGULATION OF TYPE I INTERFERON PRODUCTION	0.0033	5	37
GO:0001659	TEMPERATURE HOMEOSTASIS	0.0033	5	37
GO:0002456	T CELL MEDIATED IMMUNITY	0.0034	6	62
GO:0060760	POSITIVE REGULATION OF RESPONSE TO CYTOKINE STIMULUS	0.0036	4	19
GO:0032728	POSITIVE REGULATION OF INTERFERON-BETA PRODUCTION	0.0036	4	19
GO:0050704	REGULATION OF INTERLEUKIN-1 SECRETION	0.0036	4	19

GO:0017171	SERINE HYDROLASE ACTIVITY	0.0041	7	95
GO:0050870	POSITIVE REGULATION OF T CELL ACTIVATION	0.0041	8	130
GO:0050715	POSITIVE REGULATION OF CYTOKINE SECRETION	0.0043	6	65
GO:2000403	POSITIVE REGULATION OF LYMPHOCYTE MIGRATION	0.0043	4	20
GO:0001916	POSITIVE REGULATION OF T CELL MEDIATED CYTOTOXICITY	0.0043	4	20
GO:0050702	INTERLEUKIN-1 BETA SECRETION	0.0043	4	20
GO:0032731	POSITIVE REGULATION OF INTERLEUKIN-1 BETA PRODUCTION	0.0043	4	20
GO:0032611	INTERLEUKIN-1 BETA PRODUCTION	0.0045	5	40
GO:0044003	MODIFICATION BY SYMBIONT OF HOST MORPHOLOGY OR PHYSIOLOGY	0.0052	4	21
GO:0072676	LYMPHOCYTE MIGRATION	0.0056	5	42
GO:0002720	POSITIVE REGULATION OF CYTOKINE PRODUCTION INVOLVED IN IMMUNE RESPONSE	0.0061	4	22
GO:0032732	POSITIVE REGULATION OF INTERLEUKIN-1 PRODUCTION	0.0061	4	22
GO:0043900	REGULATION OF MULTI-ORGANISM PROCESS	0.0062	9	180
GO:0031343	POSITIVE REGULATION OF CELL KILLING	0.0062	5	43
GO:0051251	POSITIVE REGULATION OF LYMPHOCYTE ACTIVATION	0.0062	9	180
GO:0032755	POSITIVE REGULATION OF INTERLEUKIN-6 PRODUCTION	0.0062	5	43
GO:0051607	DEFENSE RESPONSE TO VIRUS	0.0067	7	104
GO:0002709	REGULATION OF T CELL MEDIATED IMMUNITY	0.0068	5	44
GO:0032481	POSITIVE REGULATION OF TYPE I INTERFERON PRODUCTION	0.0070	4	23
GO:0002828	REGULATION OF TYPE 2 IMMUNE RESPONSE	0.0070	4	23
GO:0050701	INTERLEUKIN-1 SECRETION	0.0070	4	23
GO:0016064	IMMUNOGLOBULIN MEDIATED IMMUNE RESPONSE	0.0075	6	73
GO:0032612	INTERLEUKIN-1 PRODUCTION	0.0081	5	46
GO:0001914	REGULATION OF T CELL MEDIATED CYTOTOXICITY	0.0082	4	24
GO:0019724	B CELL MEDIATED IMMUNITY	0.0084	6	75
GO:0006874	CELLULAR CALCIUM ION HOMEOSTASIS	0.0084	11	283
GO:0002526	ACUTE INFLAMMATORY RESPONSE	0.0084	6	75
GO:0002577	REGULATION OF ANTIGEN PROCESSING AND PRESENTATION	0.0107	3	10
GO:0050830	DEFENSE RESPONSE TO GRAM-POSITIVE BACTERIUM	0.0107	5	49
GO:0031620	REGULATION OF FEVER GENERATION	0.0107	3	10
GO:0061081	POSITIVE REGULATION OF MYELOID LEUKOCYTE CYTOKINE PRODUCTION INVOLVED IN IMMUNE RESPONSE	0.0107	3	10
GO:0030881	BETA-2-MICROGLOBULIN BINDING	0.0107	3	10
GO:0005581	COLLAGEN	0.0108	4	26
GO:0055074	CALCIUM ION HOMEOSTASIS	0.0117	11	295
GO:0072503	CELLULAR DIVALENT INORGANIC CATION HOMEOSTASIS	0.0120	11	296
GO:0005501	RETINOID BINDING	0.0123	4	27
GO:0031341	REGULATION OF CELL KILLING	0.0124	5	51
GO:0002440	PRODUCTION OF MOLECULAR MEDIATOR OF	0.0126	7	117

	IMMUNE RESPONSE			
GO:0002274	MYELOID LEUKOCYTE ACTIVATION	0.0132	7	118
GO:0002221	PATTERN RECOGNITION RECEPTOR SIGNALING PATHWAY	0.0135	6	83
GO:0060333	INTERFERON-GAMMA-MEDIATED SIGNALING PATHWAY	0.0135	3	11
GO:0002702	POSITIVE REGULATION OF PRODUCTION OF MOLECULAR MEDIATOR OF IMMUNE RESPONSE	0.0135	4	28
GO:0005126	CYTOKINE RECEPTOR BINDING	0.0135	9	204
GO:0030656	REGULATION OF VITAMIN METABOLIC PROCESS	0.0135	3	11
GO:2000401	REGULATION OF LYMPHOCYTE MIGRATION	0.0135	4	28
GO:0031652	POSITIVE REGULATION OF HEAT GENERATION	0.0135	3	11
GO:0042092	TYPE 2 IMMUNE RESPONSE	0.0135	4	28
GO:0032642	REGULATION OF CHEMOKINE PRODUCTION	0.0141	5	53
GO:0022407	REGULATION OF CELL-CELL ADHESION	0.0151	6	85
GO:0032675	REGULATION OF INTERLEUKIN-6 PRODUCTION	0.0159	6	86
GO:0002758	INNATE IMMUNE RESPONSE-ACTIVATING SIGNAL TRANSDUCTION	0.0159	6	86
GO:0044706	MULTI-MULTICELLULAR ORGANISM PROCESS	0.0160	7	123
GO:0050792	REGULATION OF VIRAL PROCESS	0.0168	6	87
GO:0019840	ISOPRENOID BINDING	0.0169	4	30
GO:0060338	REGULATION OF TYPE I INTERFERON-MEDIATED SIGNALING PATHWAY	0.0169	3	12
GO:0010818	T CELL CHEMOTAXIS	0.0169	3	12
GO:0010935	REGULATION OF MACROPHAGE CYTOKINE PRODUCTION	0.0169	3	12
GO:0045089	POSITIVE REGULATION OF INNATE IMMUNE RESPONSE	0.0172	7	125
GO:0032635	INTERLEUKIN-6 PRODUCTION	0.0174	6	88
GO:0044420	EXTRACELLULAR MATRIX PART	0.0187	7	127
GO:0032602	CHEMOKINE PRODUCTION	0.0187	5	57
GO:0032648	REGULATION OF INTERFERON-BETA PRODUCTION	0.0187	4	31
GO:0071622	REGULATION OF GRANULOCYTE CHEMOTAXIS	0.0187	4	31
GO:0008201	HEPARIN BINDING	0.0192	6	90
GO:0002708	POSITIVE REGULATION OF LYMPHOCYTE MEDIATED IMMUNITY	0.0199	5	58
GO:0048525	NEGATIVE REGULATION OF VIRAL PROCESS	0.0199	5	58
GO:0032651	REGULATION OF INTERLEUKIN-1 BETA PRODUCTION	0.0202	4	32
GO:0045059	POSITIVE THYMIC T CELL SELECTION	0.0202	3	13
GO:0051084	'DE NOVO' POSTTRANSLATIONAL PROTEIN FOLDING	0.0202	3	13
GO:0001972	RETINOIC ACID BINDING	0.0202	3	13
GO:0010934	MACROPHAGE CYTOKINE PRODUCTION	0.0202	3	13
GO:0032722	POSITIVE REGULATION OF CHEMOKINE PRODUCTION	0.0202	4	32
GO:0006458	'DE NOVO' PROTEIN FOLDING	0.0202	3	13
GO:0031650	REGULATION OF HEAT GENERATION	0.0202	3	13
GO:0002705	POSITIVE REGULATION OF LEUKOCYTE MEDIATED IMMUNITY	0.0207	5	59
GO:0002218	ACTIVATION OF INNATE IMMUNE RESPONSE	0.0216	6	93

GO:0002706	REGULATION OF LYMPHOCYTE MEDIATED IMMUNITY	0.0216	6	93
GO:0032608	INTERFERON-BETA PRODUCTION	0.0224	4	33
GO:0043901	NEGATIVE REGULATION OF MULTI-ORGANISM PROCESS	0.0227	6	94
GO:0030141	SECRETORY GRANULE	0.0231	10	274
GO:0035455	RESPONSE TO INTERFERON-ALPHA	0.0247	3	14
GO:0001913	T CELL MEDIATED CYTOTOXICITY	0.0248	4	34
GO:0005578	PROTEINACEOUS EXTRACELLULAR MATRIX	0.0248	8	179
GO:0004175	ENDOPEPTIDASE ACTIVITY	0.0248	8	179
GO:0050714	POSITIVE REGULATION OF PROTEIN SECRETION	0.0277	6	98
GO:0042287	MHC PROTEIN BINDING	0.0298	3	15
GO:0090036	REGULATION OF PROTEIN KINASE C SIGNALING	0.0298	3	15
GO:0015721	BILE ACID AND BILE SALT TRANSPORT	0.0298	3	15
GO:0022409	POSITIVE REGULATION OF CELL-CELL ADHESION	0.0298	4	36
GO:0032653	REGULATION OF INTERLEUKIN-10 PRODUCTION	0.0298	4	36
GO:0001912	POSITIVE REGULATION OF LEUKOCYTE MEDIATED CYTOTOXICITY	0.0298	4	36
GO:0042089	CYTOKINE BIOSYNTHETIC PROCESS	0.0300	6	100
GO:0032652	REGULATION OF INTERLEUKIN-1 PRODUCTION	0.0329	4	37
GO:0032760	POSITIVE REGULATION OF TUMOR NECROSIS FACTOR PRODUCTION	0.0364	4	38
GO:0042107	CYTOKINE METABOLIC PROCESS	0.0365	6	104
GO:0007204	POSITIVE REGULATION OF CYTOSOLIC CALCIUM ION CONCENTRATION	0.0365	8	191
GO:0032613	INTERLEUKIN-10 PRODUCTION	0.0398	4	39
GO:0046596	REGULATION OF VIRAL ENTRY INTO HOST CELL	0.0418	3	17
GO:0090023	POSITIVE REGULATION OF NEUTROPHIL CHEMOTAXIS	0.0418	3	17
GO:0005839	PROTEASOME CORE COMPLEX	0.0418	3	17
GO:0032655	REGULATION OF INTERLEUKIN-12 PRODUCTION	0.0430	4	40
GO:0042116	MACROPHAGE ACTIVATION	0.0430	4	40
GO:0050840	EXTRACELLULAR MATRIX BINDING	0.0469	4	41
GO:0031663	LIPOPOLYSACCHARIDE-MEDIATED SIGNALING PATHWAY	0.0469	4	41
GO:0019048	MODULATION BY VIRUS OF HOST MORPHOLOGY OR PHYSIOLOGY	0.0484	3	18
GO:0061082	MYELOID LEUKOCYTE CYTOKINE PRODUCTION	0.0484	3	18
GO:1902624	POSITIVE REGULATION OF NEUTROPHIL MIGRATION	0.0484	3	18
GO:0002700	REGULATION OF PRODUCTION OF MOLECULAR MEDIATOR OF IMMUNE RESPONSE	0.0487	5	73
GO:0002718	REGULATION OF CYTOKINE PRODUCTION INVOLVED IN IMMUNE RESPONSE	0.0503	4	42
GO:0032615	INTERLEUKIN-12 PRODUCTION	0.0503	4	42
GO:0051048	NEGATIVE REGULATION OF SECRETION	0.0505	7	155
GO:0051928	POSITIVE REGULATION OF CALCIUM ION TRANSPORT	0.0508	5	74
GO:2000106	REGULATION OF LEUKOCYTE APOPTOTIC PROCESS	0.0508	5	74
GO:0043271	NEGATIVE REGULATION OF ION TRANSPORT	0.0538	5	75
GO:0051222	POSITIVE REGULATION OF PROTEIN TRANSPORT	0.0540	8	205

GO:0071624	POSITIVE REGULATION OF GRANULOCYTE CHEMOTAXIS	0.0552	3	19
GO:0001910	REGULATION OF LEUKOCYTE MEDIATED CYTOTOXICITY	0.0583	4	44
GO:2000107	NEGATIVE REGULATION OF LEUKOCYTE APOPTOTIC PROCESS	0.0583	4	44
GO:0001664	G-PROTEIN COUPLED RECEPTOR BINDING	0.0622	8	210
GO:0043902	POSITIVE REGULATION OF MULTI-ORGANISM PROCESS	0.0630	4	45
GO:0051924	REGULATION OF CALCIUM ION TRANSPORT	0.0632	7	162
GO:0002703	REGULATION OF LEUKOCYTE MEDIATED IMMUNITY	0.0643	6	118
GO:0051480	CYTOSOLIC CALCIUM ION HOMEOSTASIS	0.0652	8	212
GO:1902107	POSITIVE REGULATION OF LEUKOCYTE DIFFERENTIATION	0.0667	6	119
GO:0032680	REGULATION OF TUMOR NECROSIS FACTOR PRODUCTION	0.0691	5	80
GO:0007565	FEMALE PREGNANCY	0.0691	5	80
GO:0032897	NEGATIVE REGULATION OF VIRAL TRANSCRIPTION	0.0715	3	21
GO:0032735	POSITIVE REGULATION OF INTERLEUKIN-12 PRODUCTION	0.0715	3	21
GO:0032640	TUMOR NECROSIS FACTOR PRODUCTION	0.0720	5	81
GO:0001906	CELL KILLING	0.0720	5	81
GO:0071706	TUMOR NECROSIS FACTOR SUPERFAMILY CYTOKINE PRODUCTION	0.0801	5	83
GO:0043368	POSITIVE T CELL SELECTION	0.0804	3	22
GO:0046718	VIRAL ENTRY INTO HOST CELL	0.0804	3	22
GO:0090022	REGULATION OF NEUTROPHIL CHEMOTAXIS	0.0804	3	22
GO:0005770	LATE ENDOSOME	0.0899	6	127
GO:0048246	MACROPHAGE CHEMOTAXIS	0.0903	3	23
GO:0045061	THYMIC T CELL SELECTION	0.0903	3	23
GO:0045824	NEGATIVE REGULATION OF INNATE IMMUNE RESPONSE	0.0903	3	23
GO:1902622	REGULATION OF NEUTROPHIL MIGRATION	0.0903	3	23
GO:0030260	ENTRY INTO HOST CELL	0.0990	3	24
GO:0052192	MOVEMENT IN ENVIRONMENT OF OTHER ORGANISM INVOLVED IN SYMBIOTIC INTERACTION	0.0990	3	24
GO:0002367	CYTOKINE PRODUCTION INVOLVED IN IMMUNE RESPONSE	0.0990	4	52
GO:0044409	ENTRY INTO HOST	0.0990	3	24
GO:0051806	ENTRY INTO CELL OF OTHER ORGANISM INVOLVED IN SYMBIOTIC INTERACTION	0.0990	3	24
GO:0051828	ENTRY INTO OTHER ORGANISM INVOLVED IN SYMBIOTIC INTERACTION	0.0990	3	24
GO:0061077	CHAPERONE-MEDIATED PROTEIN FOLDING	0.0990	3	24
GO:0052126	MOVEMENT IN HOST ENVIRONMENT	0.0990	3	24
GO:0019058	VIRAL LIFE CYCLE	0.0990	3	24
GO:0002675	POSITIVE REGULATION OF ACUTE INFLAMMATORY RESPONSE	0.0990	3	24
