# Supplementary material for: Different genotypes of Trypanosoma cruzi produce distinctive placental environment genetic response in chronic experimental infection
Source: PLoS Negl Trop Dis. 2017 Mar 8;11(3):e0005436. doi: 10.1371/journal.pntd.0005436 (PMC5358786; doi:10.1371/journal.pntd.0005436)
Supplement: S5 Table — (PDF) [file pntd.0005436.s005.pdf]

S5 Table. GO-Biological Process with FDR.q.val < 0.05 found by GSEA analysis in K98 group.

NAME	SIZE	NES	FDR.q.val
PROTEOLYSIS	171	-2.956	0.000
CELLULAR CARBOHYDRATE METABOLIC PROCESS	116	-3.418	0.000
CARBOHYDRATE METABOLIC PROCESS	162	-3.550	0.000
CELLULAR LIPID METABOLIC PROCESS	218	-3.646	0.000
LIPID METABOLIC PROCESS	275	-3.794	0.000
MEMBRANE LIPID METABOLIC PROCESS	88	-2.915	0.000
REGULATION OF TRANSCRIPTION	493	-3.019	0.000
PROTEOGLYCAN METABOLIC PROCESS	20	-2.898	0.000
NERVOUS SYSTEM DEVELOPMENT	362	-2.796	0.000
AMINE METABOLIC PROCESS	131	-2.776	0.000
MUSCLE DEVELOPMENT	91	-2.768	0.001
ALCOHOL METABOLIC PROCESS	80	-2.694	0.001
REGULATION OF RNA METABOLIC PROCESS	404	-2.666	0.001
NEUROLOGICAL SYSTEM PROCESS	346	-2.661	0.001
CARBOHYDRATE BIOSYNTHETIC PROCESS	45	-2.697	0.001
REGULATION OF TRANSCRIPTIONDNA DEPENDENT	399	-2.672	0.001
LIPID BIOSYNTHETIC PROCESS	89	-2.638	0.001
SULFUR COMPOUND BIOSYNTHETIC PROCESS	16	-2.611	0.001
POST TRANSLATIONAL PROTEIN MODIFICATION	434	-2.589	0.002
STEROID METABOLIC PROCESS	59	-2.589	0.002
NITROGEN COMPOUND METABOLIC PROCESS	144	-2.541	0.002
STEROID BIOSYNTHETIC PROCESS	22	-2.558	0.002
NEGATIVE REGULATION OF TRANSCRIPTION	157	-2.527	0.002
RESPONSE TO WOUNDING	169	2.612	0.002
REGULATION OF TRANSCRIPTION FROM RNA POLYMERASE II PROMOTER	251	-2.496	0.002
LOCOMOTORY BEHAVIOR	76	2.648	0.003
ANATOMICAL STRUCTURE MORPHOGENESIS	349	-2.468	0.003
ION TRANSPORT	170	-2.465	0.003
DI TRI VALENT INORGANIC CATION TRANSPORT	31	-2.408	0.004
CELL CELL SIGNALING	365	-2.416	0.004
TRANSCRIPTION FROM RNA POLYMERASE II PROMOTER	404	-2.410	0.004
CATION TRANSPORT	136	-2.379	0.005
CALCIUM ION TRANSPORT	26	-2.371	0.005
CARBOXYLIC ACID TRANSPORT	34	-2.361	0.006
REGULATION OF BIOLOGICAL QUALITY	372	-2.324	0.007
SPHINGOLIPID METABOLIC PROCESS	25	-2.314	0.008
NEGATIVE REGULATION OF METABOLIC PROCESS	221	-2.291	0.008
CELL MIGRATION	82	-2.301	0.008

NEGATIVE REGULATION OF CELLULAR METABOLIC PROCESS	219	-2.295	0.008
GENERATION OF NEURONS	79	-2.281	0.008
EXCRETION	36	-2.267	0.009
ORGANIC ACID TRANSPORT	35	-2.267	0.009
NEGATIVE REGULATION OF NUCLEOBASENUCLEOSIDENUCLEOTIDE AND NUCLEIC ACID METABOLIC PROCESS	175	-2.258	0.009
RESPONSE TO EXTERNAL STIMULUS	275	2.465	0.010
PHOSPHOLIPID METABOLIC PROCESS	64	-2.237	0.010
REGULATION OF BLOOD PRESSURE	20	-2.215	0.012
CARBOXYLIC ACID METABOLIC PROCESS	157	-2.185	0.012
SYNAPTIC TRANSMISSION	166	-2.187	0.012
CELLULAR MORPHOGENESIS DURING DIFFERENTIATION	45	-2.189	0.013
ORGANIC ACID METABOLIC PROCESS	159	-2.192	0.013
HORMONE METABOLIC PROCESS	28	-2.197	0.013
ESTABLISHMENT OF PROTEIN LOCALIZATION	173	-2.177	0.013
SENSORY PERCEPTION	166	-2.194	0.013
DEFENSE RESPONSE	212	2.387	0.013
TRANSMISSION OF NERVE IMPULSE	180	-2.172	0.013
PROTEIN TARGETING	101	-2.163	0.014
NEURON DIFFERENTIATION	72	-2.148	0.015
SKELETAL DEVELOPMENT	95	-2.141	0.015
AXONOGENESIS	41	-2.128	0.017
ECTODERM DEVELOPMENT	73	2.262	0.017
ORGAN MORPHOGENESIS	136	-2.122	0.017
INFLAMMATORY RESPONSE	114	2.279	0.018
POSITIVE REGULATION OF METABOLIC PROCESS	209	-2.111	0.018
CELL PROLIFERATION GO 0008283	457	-2.104	0.019
BRAIN DEVELOPMENT	48	-2.099	0.019
BEHAVIOR	129	2.291	0.019
MACROMOLECULE LOCALIZATION	212	-2.075	0.022
REGULATION OF G PROTEIN COUPLED RECEPTOR PROTEIN SIGNALING PATHWAY	22	-2.062	0.024
VITAMIN METABOLIC PROCESS	16	-2.056	0.024
GLYCOLIPID METABOLIC PROCESS	16	-2.056	0.024
NEURITE DEVELOPMENT	49	-2.045	0.024
POSITIVE REGULATION OF CELLULAR METABOLIC PROCESS	203	-2.043	0.024
PROTEIN LOCALIZATION	194	-2.027	0.026
MONOCARBOXYLIC ACID METABOLIC PROCESS	75	-2.009	0.027
GLYCOPROTEIN METABOLIC PROCESS	82	-2.013	0.027
SECRETION	164	-2.015	0.027
G PROTEIN COUPLED RECEPTOR PROTEIN SIGNALING PATHWAY	301	-2.016	0.027
LIPID TRANSPORT	27	-2.009	0.027
NEUROGENESIS	88	-2.016	0.027

INTRACELLULAR PROTEIN TRANSPORT	135	-2.017	0.028
SULFUR METABOLIC PROCESS	35	-1.997	0.028
NEGATIVE REGULATION OF RNA METABOLIC PROCESS	107	-2.000	0.028
BIOSYNTHETIC PROCESS	414	-1.989	0.028
INTRACELLULAR TRANSPORT	252	-1.992	0.028
NEGATIVE REGULATION OF TRANSCRIPTION DNA DEPENDENT	107	-1.994	0.028
AMINE TRANSPORT	34	-1.982	0.029
PROTEIN TRANSPORT	144	-1.979	0.029
STRIATED MUSCLE DEVELOPMENT	40	-1.971	0.030
TRANSMEMBRANE RECEPTOR PROTEIN SERINE THREONINE KINASE SIGNALING PATHWAY	46	-1.966	0.031
CATABOLIC PROCESS	207	-1.959	0.032
MACROMOLECULE CATABOLIC PROCESS	124	-1.940	0.035
EPIDERMIS DEVELOPMENT	66	2.130	0.036
RESPONSE TO STRESS	450	-1.932	0.036
PROTEIN AMINO ACID PHOSPHORYLATION	248	-1.929	0.037
POSITIVE REGULATION OF TRANSCRIPTION	127	-1.920	0.038
CELL MATRIX ADHESION	36	-1.917	0.038
METAL ION TRANSPORT	110	-1.908	0.040
CELLULAR CATABOLIC PROCESS	199	-1.904	0.041
REGULATION OF MOLECULAR FUNCTION	299	-1.895	0.042
CELL CELL ADHESION	80	-1.878	0.045
CELLULAR MACROMOLECULE CATABOLIC PROCESS	95	-1.883	0.045
ESTABLISHMENT OF CELLULAR LOCALIZATION	318	-1.878	0.045
REGULATION OF DEVELOPMENTAL PROCESS	400	-1.874	0.045
PHOSPHOINOSITIDE METABOLIC PROCESS	25	-1.879	0.045
POSITIVE REGULATION OF CATALYTIC ACTIVITY	151	-1.871	0.045
REGULATION OF CELL PROLIFERATION	274	-1.861	0.048
IMMUNE SYSTEM PROCESS	273	2.047	0.049
PROTEIN PROCESSING	44	-1.854	0.050
HOMEOSTATIC_PROCESS	186	-1.852	0.050
