# Supplementary material for: Different genotypes of Trypanosoma cruzi produce distinctive placental environment genetic response in chronic experimental infection
Source: PLoS Negl Trop Dis. 2017 Mar 8;11(3):e0005436. doi: 10.1371/journal.pntd.0005436 (PMC5358786; doi:10.1371/journal.pntd.0005436)
Supplement: S6 Table — (PDF) [file pntd.0005436.s006.pdf]

S6 Table. GO-Biological Process with FDR.q.val < 0.05 found by GSEA analysis in VD group.

NAME	SIZE	NES	FDR.q.val
PROTEOLYSIS	171	-2 .885	0 .000
CELLULAR CARBOHYDRATE METABOLIC PROCESS	116	-3 .240	0 .000
CARBOHYDRATE METABOLIC PROCESS	162	-3 .455	0 .000
CELLULAR LIPID METABOLIC PROCESS	218	-3 .217	0 .000
LIPID METABOLIC PROCESS	275	-3 .417	0 .000
RESPONSE TO WOUNDING	169	4 .080	0 .000
LOCOMOTORY BEHAVIOR	76	4 .008	0 .000
RESPONSE TO EXTERNAL STIMULUS	275	3 .993	0 .000
DEFENSE RESPONSE	212	4 .639	0 .000
INFLAMMATORY RESPONSE	114	4 .247	0 .000
BEHAVIOR	129	3 .895	0 .000
BIOSYNTHETIC PROCESS	414	-3 .787	0 .000
RESPONSE TO STRESS	450	3 .030	0 .000
IMMUNE SYSTEM PROCESS	273	4 .152	0 .000
IMMUNE RESPONSE	195	3 .568	0 .000
RESPONSE TO CHEMICAL STIMULUS	260	2 .987	0 .000
CELLULAR BIOSYNTHETIC PROCESS	280	-3 .039	0 .000
CELLULAR DEFENSE RESPONSE	41	3 .287	0 .000
PROTEIN FOLDING	54	-2 .781	0 .000
CELLULAR LOCALIZATION	335	-2 .784	0 .000
ORGANIC ACID METABOLIC PROCESS	159	-2 .786	0 .000
CELLULAR COMPONENT ASSEMBLY	270	-2 .788	0 .000
INTRACELLULAR TRANSPORT	252	-2 .800	0 .000
VESICLE MEDIATED TRANSPORT	166	-2 .838	0 .000
NUCLEOBASENUCLEOSIDE AND NUCLEOTIDE METABOLIC PROCESS	48	-2 .803	0 .000
ESTABLISHMENT OF CELLULAR LOCALIZATION	318	-2 .807	0 .000
MACROMOLECULE BIOSYNTHETIC PROCESS	281	-2 .817	0 .000
LIPID BIOSYNTHETIC PROCESS	89	-2 .826	0 .000
MACROMOLECULAR COMPLEX ASSEMBLY	253	-2 .826	0 .000
CARBOXYLIC ACID METABOLIC PROCESS	157	-2 .831	0 .000
NITROGEN COMPOUND METABOLIC PROCESS	144	-2 .836	0 .000
MEMBRANE ORGANIZATION AND BIOGENESIS	120	-2 .700	0 .000
CARBOHYDRATE BIOSYNTHETIC PROCESS	45	-2 .656	0 .001
AMINE METABOLIC PROCESS	131	-2 .611	0 .001
SECRETION	164	-2 .577	0 .001
RNA PROCESSING	136	-2 .583	0 .001
MEMBRANE FUSION	28	-2 .536	0 .001
PROTEIN RNA COMPLEX ASSEMBLY	49	-2 .524	0 .002
GOLGI VESICLE TRANSPORT	43	-2 .518	0 .002

CELL CYCLE GO 0007049	281	-2 .501	0 .002
MULTI ORGANISM PROCESS	122	2 .704	0 .002
MEMBRANE LIPID METABOLIC PROCESS	88	-2 .468	0 .003
G PROTEIN COUPLED RECEPTOR PROTEIN SIGNALING PATHWAY	301	2 .683	0 .003
RESPONSE TO ENDOGENOUS STIMULUS	178	-2 .461	0 .003
RESPONSE TO OTHER ORGANISM	63	2 .652	0 .003
ECTODERM DEVELOPMENT	73	2 .627	0 .003
NUCLEOTIDE METABOLIC PROCESS	38	-2 .389	0 .004
REGULATION OF TRANSCRIPTION	493	-2 .373	0 .005
COAGULATION	37	-2 .358	0 .005
EPIDERMIS DEVELOPMENT	66	2 .541	0 .005
ESTABLISHMENT OF PROTEIN LOCALIZATION	173	-2 .345	0 .005
CARBOXYLIC ACID TRANSPORT	34	-2 .323	0 .006
NERVOUS SYSTEM DEVELOPMENT	362	-2 .325	0 .006
ION HOMEOSTASIS	116	2 .519	0 .006
COENZYME METABOLIC PROCESS	35	-2 .311	0 .006
MACROMOLECULE LOCALIZATION	212	-2 .303	0 .006
BLOOD COAGULATION	37	-2 .297	0 .006
ER TO GOLGI VESICLE MEDIATED TRANSPORT	17	-2 .294	0 .006
G1 S TRANSITION OF MITOTIC CELL CYCLE	26	-2 .287	0 .006
LEUKOCYTE ACTIVATION	55	2 .485	0 .006
ORGANIC ACID TRANSPORT	35	-2 .266	0 .007
CELL CELL SIGNALING	365	2 .459	0 .007
SECRETION BY CELL	105	-2 .259	0 .007
CELL ACTIVATION	59	2 .426	0 .008
PROTEIN COMPLEX ASSEMBLY	159	-2 .242	0 .008
RESPONSE TO VIRUS	39	2 .408	0 .008
RESPONSE TO BIOTIC STIMULUS	94	2 .399	0 .008
RESPONSE TO DNA DAMAGE STIMULUS	145	-2 .233	0 .008
TRANSLATIONAL INITIATION	31	-2 .236	0 .008
CELLULAR HOMEOSTASIS	132	2 .376	0 .008
HOMEOSTATIC PROCESS	186	2 .409	0 .009
PHOSPHOLIPID BIOSYNTHETIC PROCESS	35	-2 .224	0 .009
LYMPHOCYTE ACTIVATION	51	2 .377	0 .009
REGULATION OF APOPTOSIS	310	-2 .198	0 .010
PROTEIN KINASE CASCADE	263	2 .338	0 .010
BIOPOLYMER CATABOLIC PROCESS	105	-2 .201	0 .010
REGULATION OF PROGRAMMED CELL DEATH	311	-2 .181	0 .011
CHEMICAL HOMEOSTASIS	139	2 .311	0 .011
PROTEIN LOCALIZATION	194	-2 .168	0 .011
REGULATION OF CELLULAR COMPONENT ORGANIZATION AND BIOGENESIS	110	-2 .153	0 .012

GLYCEROPHOSPHOLIPID METABOLIC PROCESS	39	-2 .148	0 .013
GLYCOPROTEIN METABOLIC PROCESS	82	-2 .132	0 .013
DNA REPLICATION	84	-2 .135	0 .013
REGULATION OF RNA METABOLIC PROCESS	404	-2 .129	0 .014
HEMOSTASIS	41	-2 .113	0 .015
GLYCEROPHOSPHOLIPID BIOSYNTHETIC PROCESS	28	-2 .110	0 .015
AMINO ACID TRANSPORT	22	-2 .107	0 .015
COFACTOR METABOLIC PROCESS	51	-2 .103	0 .015
CATION HOMEOSTASIS	98	2 .246	0 .015
HEMOPOIESIS	66	2 .239	0 .015
PHOSPHORYLATION	277	2 .226	0 .015
NEGATIVE REGULATION OF DEVELOPMENTAL PROCESS	183	2 .219	0 .016
HEMOPOIETIC OR LYMPHOID ORGAN DEVELOPMENT	68	2 .186	0 .016
REGULATION OF BIOLOGICAL QUALITY	372	2 .190	0 .016
ESTABLISHMENT AND OR MAINTENANCE OF CELL POLARITY	18	2 .195	0 .016
REGULATION OF LYMPHOCYTE ACTIVATION	30	2 .199	0 .016
REGULATION OF TRANSLATIONAL INITIATION	25	-2 .085	0 .016
ACTIN FILAMENT BASED PROCESS	104	2 .163	0 .017
ACTIN CYTOSKELETON ORGANIZATION AND BIOGENESIS	95	2 .170	0 .017
CELLULAR CATION HOMEOSTASIS	95	2 .137	0 .018
CYTOKINE METABOLIC PROCESS	39	2 .140	0 .018
REGULATION OF CELL CYCLE	162	-2 .065	0 .019
VIRAL GENOME REPLICATION	17	2 .101	0 .020
CYTOKINE BIOSYNTHETIC PROCESS	38	2 .104	0 .020
REGULATION OF DEVELOPMENTAL PROCESS	400	-2 .051	0 .020
IMMUNE SYSTEM DEVELOPMENT	71	2 .085	0 .021
CELL CYCLE CHECKPOINT GO 0000075	42	-2 .033	0 .022
REGULATION OF CELL PROLIFERATION	274	-2 .034	0 .022
MYOBLAST DIFFERENTIATION	17	-2 .021	0 .022
AMINO SUGAR METABOLIC PROCESS	18	-2 .019	0 .023
CENTRAL NERVOUS SYSTEM DEVELOPMENT	117	-2 .023	0 .023
CELL PROLIFERATION GO 0008283	457	-2 .012	0 .023
CELL CYCLE PROCESS	177	-2 .024	0 .023
PROTEIN MODIFICATION BY SMALL PROTEIN CONJUGATION	39	-2 .014	0 .023
PROTEIN CATABOLIC PROCESS	60	-2 .005	0 .023
REGULATION OF TRANSCRIPTIONDNA DEPENDENT	399	-2 .007	0 .023
PROTEIN AMINO ACID N LINKED GLYCOSYLATION	27	-2 .014	0 .023
PHOSPHOLIPID METABOLIC PROCESS	64	-2 .008	0 .023
AROMATIC COMPOUND METABOLIC PROCESS	25	-1 .996	0 .023
DNA METABOLIC PROCESS	229	-1 .997	0 .023
INTRACELLULAR PROTEIN TRANSPORT	135	-1 .974	0 .024

DNA REPAIR	116	-1 .981	0 .024
CELLULAR PROTEIN CATABOLIC PROCESS	53	-1 .975	0 .024
CELLULAR MORPHOGENESIS DURING DIFFERENTIATION	45	-1 .977	0 .024
POSITIVE REGULATION OF T CELL ACTIVATION	17	2 .049	0 .024
REGULATION OF BODY FLUID LEVELS	50	-1 .978	0 .024
REPRODUCTION	217	-1 .982	0 .024
MONOCARBOXYLIC ACID METABOLIC PROCESS	75	-1 .982	0 .025
REPRODUCTIVE PROCESS	128	-1 .960	0 .025
NUCLEOTIDE BIOSYNTHETIC PROCESS	16	-1 .983	0 .025
STEROID BIOSYNTHETIC PROCESS	22	-1 .960	0 .025
UBIQUITIN CYCLE	44	-1 .966	0 .025
MACROMOLECULE CATABOLIC PROCESS	124	-1 .956	0 .025
SULFUR COMPOUND BIOSYNTHETIC PROCESS	16	-1 .964	0 .025
CATABOLIC PROCESS	207	-1 .961	0 .025
PHOSPHOINOSITIDE BIOSYNTHETIC PROCESS	22	-1 .962	0 .025
HETEROCYCLE METABOLIC PROCESS	25	-1 .949	0 .026
MEMBRANE LIPID BIOSYNTHETIC PROCESS	43	-1 .944	0 .026
NEGATIVE REGULATION OF METABOLIC PROCESS	221	-1 .945	0 .026
MITOTIC CELL CYCLE	143	-1 .937	0 .026
GENERATION OF NEURONS	79	-1 .938	0 .027
PROTEOGLYCAN METABOLIC PROCESS	20	-1 .929	0 .027
GLYCOPROTEIN BIOSYNTHETIC PROCESS	67	-1 .930	0 .027
POSITIVE REGULATION OF TRANSLATION	30	2 .023	0 .027
PHOSPHOINOSITIDE METABOLIC PROCESS	25	-1 .921	0 .028
AXONOGENESIS	41	-1 .921	0 .028
INTERPHASE	64	-1 .916	0 .028
MRNA METABOLIC PROCESS	62	-1 .917	0 .028
LEUKOCYTE DIFFERENTIATION	34	2 .013	0 .028
NEURITE DEVELOPMENT	49	-1 .922	0 .028
POSITIVE REGULATION OF CYTOKINE BIOSYNTHETIC PROCESS	23	2 .004	0 .029
NEGATIVE REGULATION OF CELLULAR METABOLIC PROCESS	219	-1 .906	0 .029
WOUND HEALING	48	-1 .907	0 .029
NEGATIVE REGULATION OF NUCLEOBASENUCLEOSIDENUCLEOTIDE AND NUCLEIC ACID METABOLIC PROCESS	175	-1 .903	0 .029
SECRETORY PATHWAY	77	-1 .896	0 .030
CELL CYCLE PHASE	157	-1 .897	0 .030
ALCOHOL METABOLIC PROCESS	80	-1 .891	0 .030
PROTEIN TRANSPORT	144	-1 .883	0 .031
PROGRAMMED CELL DEATH	390	-1 .878	0 .031
AMINO ACID AND DERIVATIVE METABOLIC PROCESS	93	-1 .883	0 .031
REGULATION OF MOLECULAR FUNCTION	299	-1 .872	0 .031
CELLULAR MACROMOLECULE CATABOLIC PROCESS	95	-1 .869	0 .031

APOPTOSIS GO	389	-1 .879	0 .031
CELLULAR CATABOLIC PROCESS	199	-1 .873	0 .031
STEROID METABOLIC PROCESS	59	-1 .883	0 .031
SULFUR METABOLIC PROCESS	35	-1 .875	0 .031
POSITIVE REGULATION OF DEVELOPMENTAL PROCESS	199	-1 .869	0 .031
ENDOSOME TRANSPORT	19	-1 .873	0 .031
TISSUE DEVELOPMENT	127	1 .979	0 .032
B CELL ACTIVATION	18	1 .971	0 .032
BRAIN DEVELOPMENT	48	-1 .858	0 .033
T CELL ACTIVATION	36	1 .963	0 .033
MRNA PROCESSING GO 0006397	52	-1 .855	0 .033
NEUROGENESIS	88	-1 .852	0 .034
EXOCYTOSIS	24	1 .955	0 .034
POSITIVE REGULATION OF LYMPHOCYTE ACTIVATION	19	1 .950	0 .034
REGULATION OF IMMUNE SYSTEM PROCESS	55	1 .943	0 .034
REGULATION OF MAP KINASE ACTIVITY	62	1 .938	0 .034
LYMPHOCYTE DIFFERENTIATION	24	1 .933	0 .035
FATTY ACID METABOLIC PROCESS	51	-1 .844	0 .035
RIBONUCLEOPROTEIN COMPLEX BIOGENESIS AND ASSEMBLY	63	-1 .837	0 .036
REGULATION OF CYTOKINE BIOSYNTHETIC PROCESS	35	1 .913	0 .037
PROTEIN AMINO ACID PHOSPHORYLATION	248	1 .915	0 .037
CELLULAR RESPIRATION	19	-1 .824	0 .038
REGULATION OF T CELL ACTIVATION	24	1 .900	0 .038
REGULATION OF HYDROLASE ACTIVITY	73	-1 .821	0 .039
PROTEIN UBIQUITINATION	37	-1 .813	0 .040
CHROMATIN ASSEMBLY OR DISASSEMBLY	26	-1 .800	0 .043
REGULATION OF BLOOD PRESSURE	20	-1 .795	0 .043
VITAMIN METABOLIC PROCESS	16	-1 .796	0 .043
GENERATION OF PRECURSOR METABOLITES AND ENERGY	112	-1 .790	0 .044
REGULATION OF MULTICELLULAR ORGANISMAL PROCESS	127	1 .861	0 .046
POST TRANSLATIONAL PROTEIN MODIFICATION	434	-1 .775	0 .047
NUCLEAR TRANSPORT	79	-1 .775	0 .047
POSITIVE REGULATION OF I KAPPAB KINASE NF KAPPAB CASCADE	76	-1 .771	0 .048
REGULATION OF CATALYTIC ACTIVITY	252	-1 .760	0 .050
