# Supplementary material for: Different genotypes of Trypanosoma cruzi produce distinctive placental environment genetic response in chronic experimental infection
Source: PLoS Negl Trop Dis. 2017 Mar 8;11(3):e0005436. doi: 10.1371/journal.pntd.0005436 (PMC5358786; doi:10.1371/journal.pntd.0005436)
Supplement: S7 Table — The table shows only the polymorphic loci. (PDF) [file pntd.0005436.s007.pdf]

S7 Table. Examples of microsatellite identification of T. cruzi isolates from maternal and placental populations in mice infected with K98 and VD genotypes. The table shows only the polymorphic loci.

			**Locus (pb)**			
***T. cruzi* strain**	**Binomial**	**Sample**	**TcTAC15**	**TcATT14**	**TcGAG10**	**TcCAA10**
K98						
	1	Maternal Blood	96/96	253/253	144/144	125/125
		Placenta	96/96	253/253	144/144	125/125
	2	Maternal Blood	96/96	253/253	144/144	125/125
		Placenta A	96/96	253/253	144/144	125/125
		Placenta B	96/96	253/253	144/144	125/125
	3	Maternal Blood	96/96	253/253	144/144	125/125
		Placenta	96/96	253/253	144/144	125/125
VD						
	1	Maternal Blood	126/129	265/265	138/144	131/155
		Placenta	126/129	265/271	138/144	131/155
	2	Maternal Blood	126/129	265/265	138/138	131/155
		Placenta A	126/129	265/271	138/144	131/155
		Placenta B	126/129	265/271	138/144	131/155
	3	Maternal Blood	126/129	265/265	138/138	131/131
		Placenta	126/129	265/271	138/144	131/155
