# Supplementary figures and images for: Different genotypes of Trypanosoma cruzi produce distinctive placental environment genetic response in chronic experimental infection
Source: PLoS Negl Trop Dis. 2017 Mar 8;11(3):e0005436. doi: 10.1371/journal.pntd.0005436 (PMC5358786; doi:10.1371/journal.pntd.0005436)

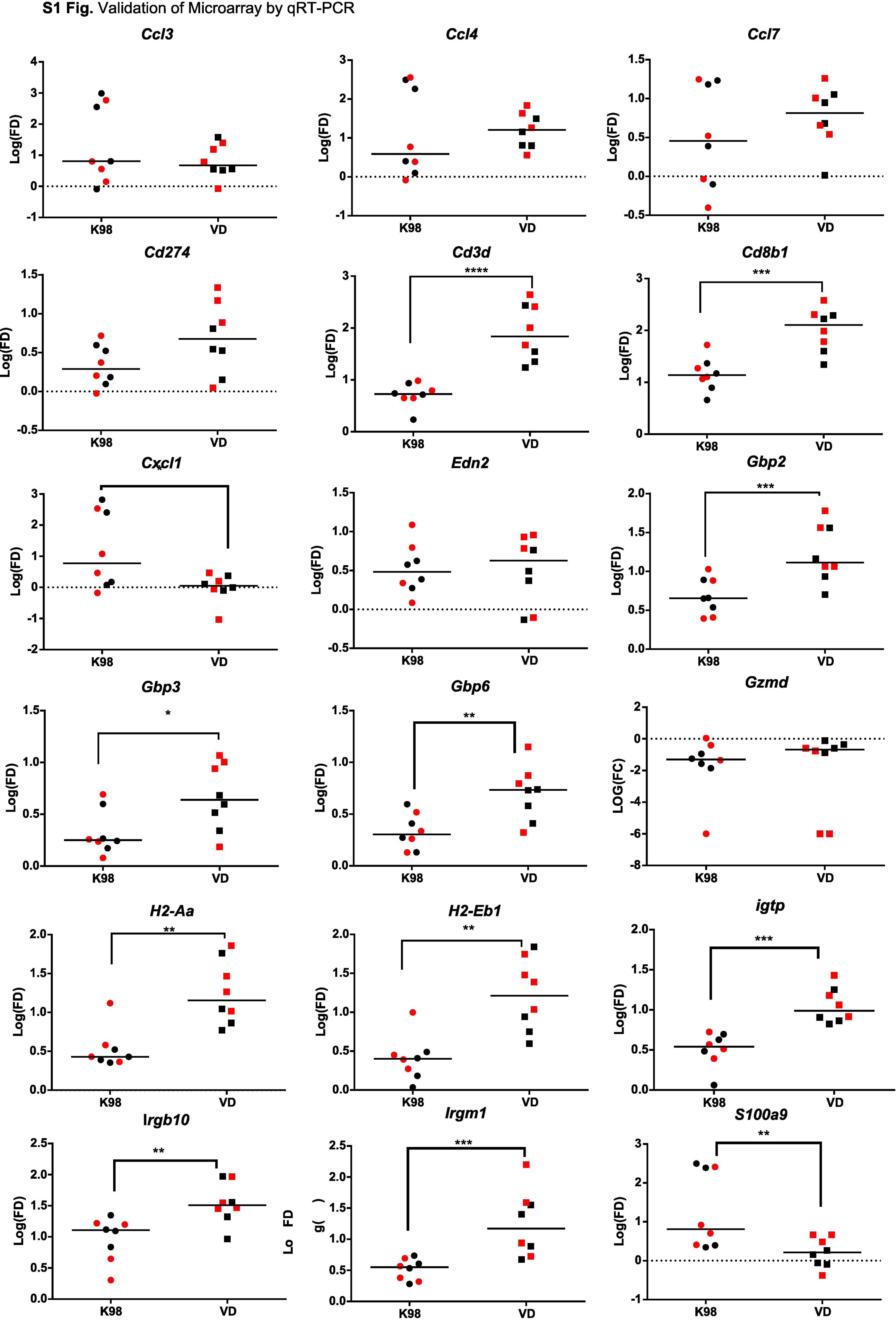

Supplement: S1 Fig — The expression levels of the selected genes were expressed as logarithms of fold change in arbitrary units (Log (FC)) normalized to Gapdh. Red symbols represent placenta samples used in microarray assay and black symbols represent independent samples. The line for each of the scatters represents the median value and significance obtained by Mann-Withney test was indicated as follows: **** p < 0.00001; *** p < 0.0001; ** p < 0.01; *p < 0.05. (TIF) [file pntd.0005436.s008.tif]

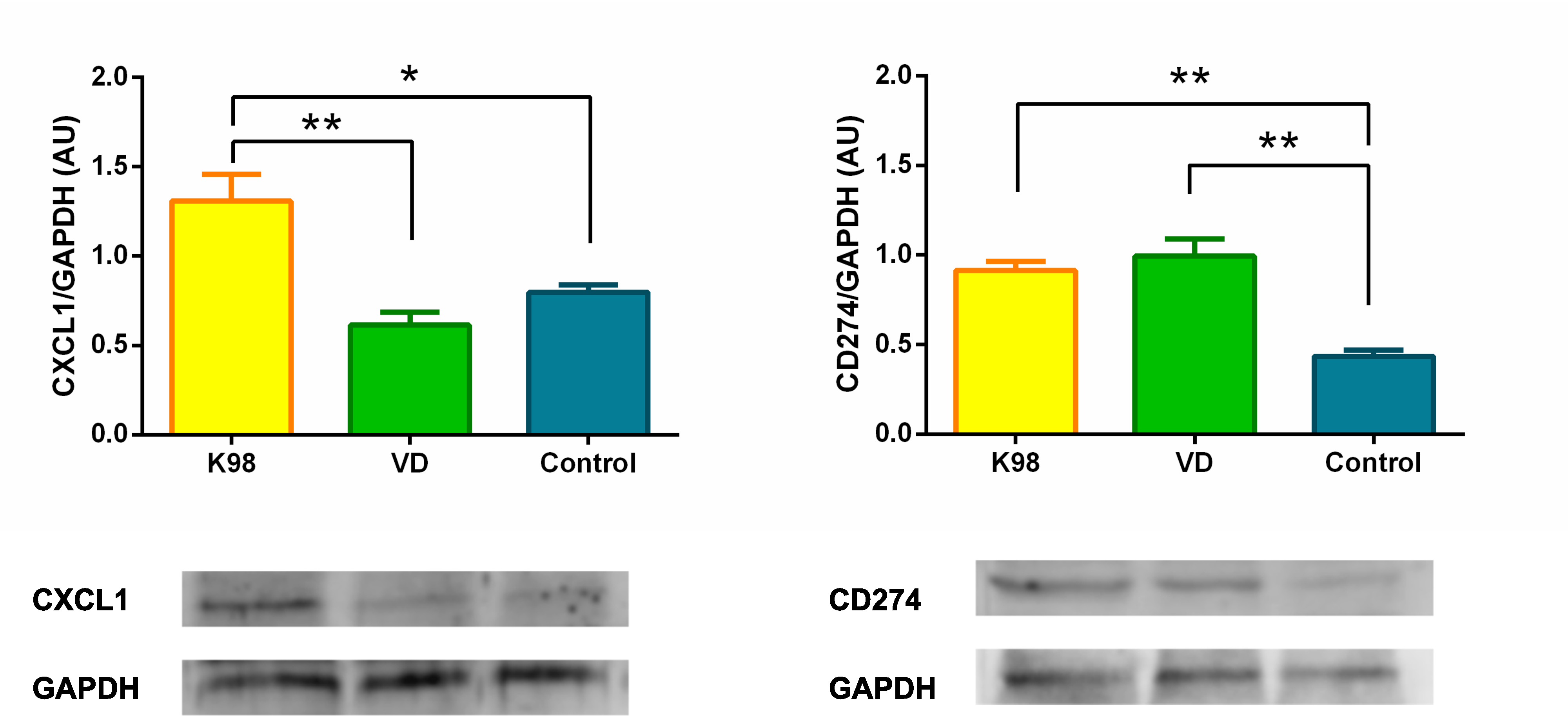

Supplement: S2 Fig — Inmunoblot analysis of CXCL1 and CD274 in placentas samples from K98, VD and control groups. Inmunoreactive protein bands were quantified by densitometry. Results are expressed as arbitrary units (AU), related to GAPDH. Statistical analysis was performed by Kruskal-Wallis with multiple comparisons. ** p < 0.01; *p < 0.05. (TIF) [file pntd.0005436.s009.tif]
